# DUSP family phosphatases in cell signaling, inflammation, and chronic diseases

**DOI:** 10.1186/s12929-026-01251-0

**Published:** 2026-05-06

**Authors:** Chia-Wen Wang, Huai-Chia Chuang, Tse-Hua Tan

**Affiliations:** Immunology Research Center, National Health Research Institutes, 35 Keyan Road, Zhunan, 35053 Taiwan

**Keywords:** Phosphatase, DUSP, MKP, Inflammation, Autoimmune diseases, Allergic diseases, Obesity, Diabetes, Metabolic diseases, Cardiovascular diseases

## Abstract

Multiple members (DUSP1–29) of dual-specificity phosphatase (DSP) family are key regulators of mitogen-activated protein kinases (MAPKs), which regulate numerous physiological responses. Eight DUSPs are also named MAPK phosphatases (MKPs). DUSP dysregulation contributes to the pathogenesis of various human inflammatory and chronic diseases. Downregulation of DUSP1, DUSP3, DUSP11, and DUSP22, as well as upregulation of DUSP4, DUSP6, and DUSP23 are involved in human autoimmune diseases. Besides autoimmune diseases, reduction of DUSP1, DUSP2, and DUSP14, as well as induction of DUSP8 contribute to the pathogenesis of allergic diseases. Additionally, decreased levels of DUSP2, DUSP11, DUSP22, and DUSP28 are associated with human inflammatory bowel diseases. Moreover, deficiency of 10 DUSPs, as well as induction of DUSP4 are associated with metabolic diseases. Downregulation of 5 DUSPs are involved in cardiovascular disease pathogenesis; in contrast, upregulation of other 5 DUSPs are correlated with cardiovascular diseases. Collectively, dysregulated DUSPs could be diagnostic biomarkers and therapeutic targets for inflammatory diseases. Due to complex expression patterns of DUSPs, it is crucial to study the regulatory mechanisms of individual DUSPs in various inflammatory and chronic diseases. In this review, we summarize the roles and regulatory mechanisms of DUSPs in human inflammatory and chronic diseases. We also discuss the potential therapeutic applications of DUSP agonists/inhibitors in human inflammatory and chronic diseases.

## Background

**Fig. 1:**
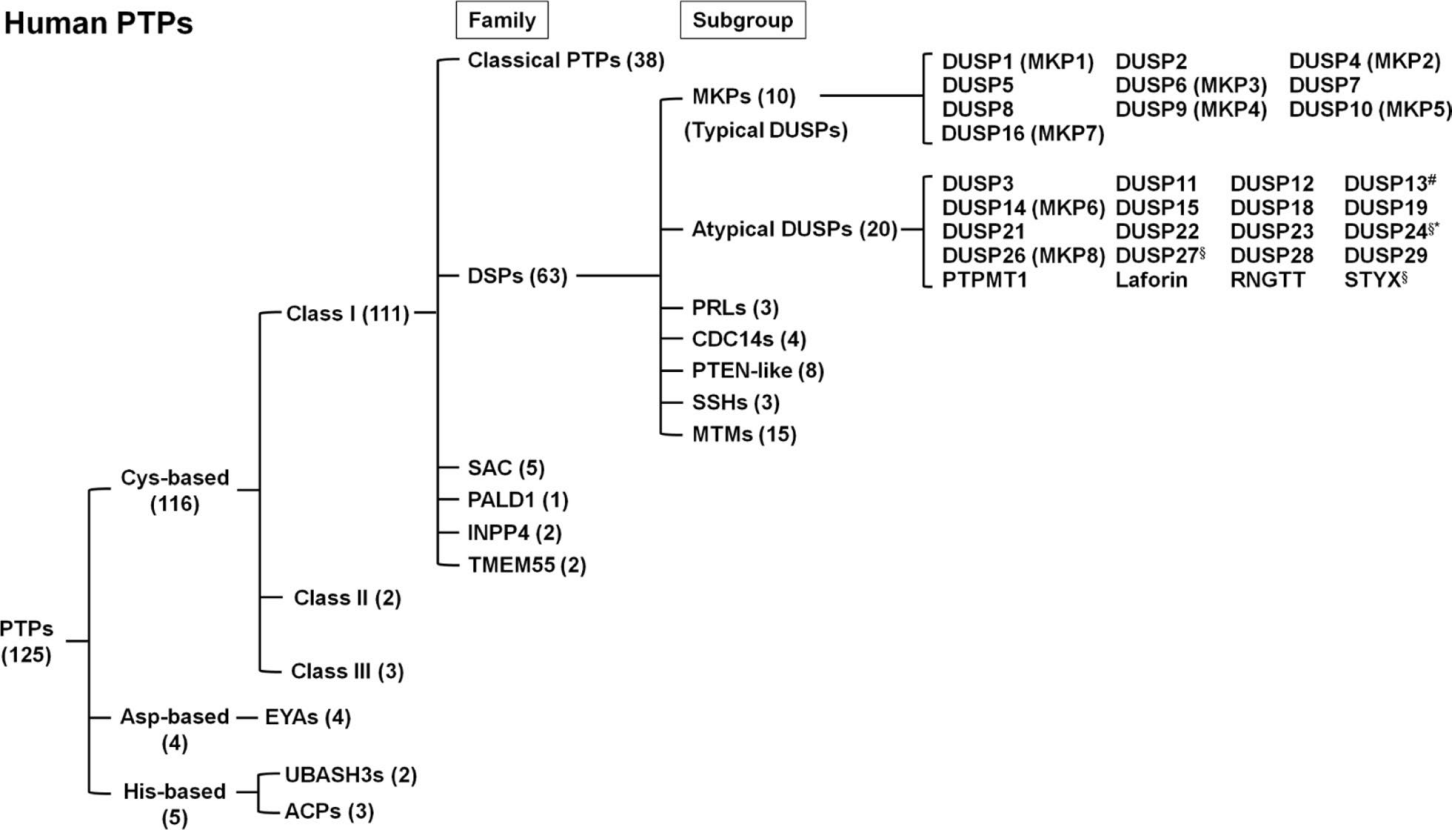
Classification of human PTPs. There are 125 identified human PTPs, 111 of them are classified as Cys-based class I PTPs. Among the human Cys-based class I PTPs, 63 PTPs are DSPs, including 10 MKPs (typical DUSPs) and 20 atypical DUSPs. The DUSP13^#^ gene encodes DUSP13A and DUSP13B isoforms, which were classified as 2 different atypical DUSPs. DUSP24^*^ does not contain the KIM, therefore is classified into atypical DUSP. However, DUSP24 was classified as MKP (typical DUSP) in some publications. DUSP24^§^, DUSP27^§^, and STYX^§^ are pseudophosphatases. *PRL* phosphatase of regenerating liver, *SSH* slingshot, *MTM* myotubularin, *PTPMT1* protein tyrosine phosphatase mitochondrial 1, *RNGTT* RNA guanylyltransferase and 5′-Phosphatase, *STYX* serine/threonine/tyrosine-interacting protein

**Fig. 2:**
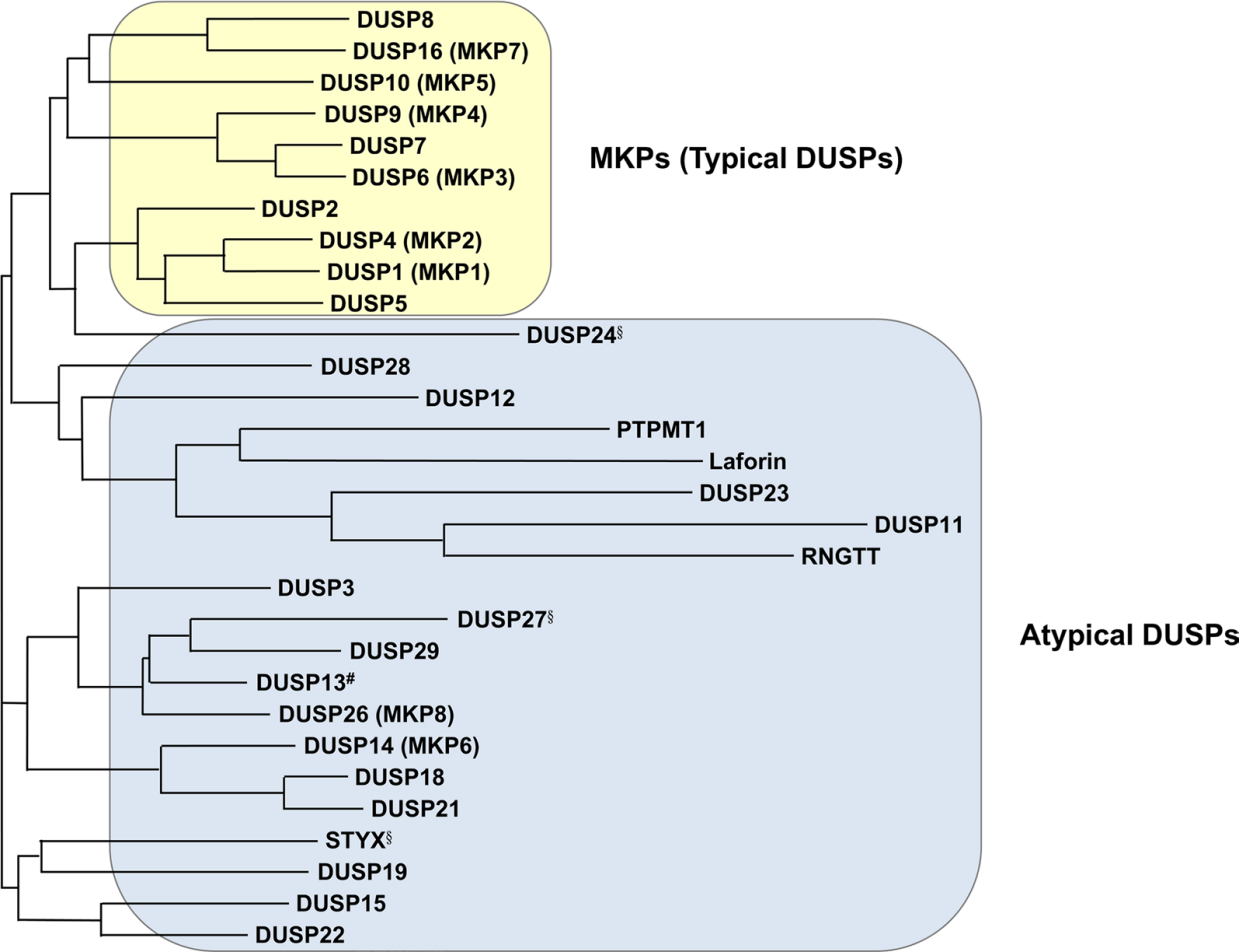
The phylogenetic tree of human MKPs (typical DUSPs) and atypical DUSPs. DUSP24^§^, DUSP27^§^, and STYX^§^ are pseudophosphatases. The DUSP13^#^ gene encodes DUSP13A and DUSP13B isoforms, and DUSP13A is shown here. The protein sequence alignment was operated by MUSCLE 5. The phylogenetic tree was generated by SIMPLE PHYLOGENY

Dual-specificity phosphatase (DSP for the family name) family belongs to cysteine-based class I protein tyrosine phosphatases (PTPs), which contain a conserved PTP signature active motif C-X_5_-R [1–6]. Notably, dual-specificity phosphatase subgroup is abbreviated as DUSP [7–9] (Fig. 1). Besides dephosphorylating tyrosine residues, DSPs also dephosphorylate serine/threonine residues due to the broader and shallower catalytic pocket than that of classical PTPs [2, 6, 10–17]. For example, PTP1B contains a 9–10 Ả deep catalytic pocket [11, 18], whereas the MKP (typical DUSP) DUSP6 contains a 5.5 Ả deep catalytic pocket [10]. Human DSP family comprises 63 phosphatases and are classified into 7 subgroups, including 10 MKPs (typical DUSPs), 20 atypical DUSPs, 3 PRLs, 4 CDC14s, 8 PTEN-like phosphatases, 3 slingshots (SSHs), and 15 myotubularins (MTMs) [5, 8, 9] (Fig. 1). The classification of MKPs (typical DUSPs, 10 members) and atypical DUSPs (20 members) are based on the presence or absence of the MAPK-interacting motif (KIM) [19–21] (Fig. 2; Tables 1 and 2). The rhodanese domain is a homolog of CDC25 catalytic domain; it is present in all MKPs (typical DUSPs) and one atypical DUSP (DUSP24) (Tables 1 and 2). KIM is present in the rhodanese domain of only MKPs (typical DUSPs) but not DUSP24. The role of KIM is to facilitate MAPK recruitment and direct MAPK’s phosphorylated residues to the phosphatase catalytic pocket of MKPs (typical DUSPs) [22]. Among these 10 MKPs (typical DUSPs) and 20 atypical DUSPs, 26 members are named as DUSPs, except 4 atypical DUSPs (PTPMT1, Laforin, RNGTT, and STYX) [8, 9] (Fig. 1; Tables 1 and 2). All 10 MKPs (typical DUSPs) downregulate and inactivate MAPK pathways by dephosphorylating the T-X-Y motif in the kinase domain of the MAPKs extracellular regulated kinase (ERK), c-Jun N-terminal kinase (JNK), and/or p38 subgroups [21, 23]. Notably, among 20 atypical DUSPs (lacking the KIM) (Table 2), DUSP3, DUSP14, and DUSP23 still can directly dephosphorylate one or more MAPKs [24–27], while other 17 atypical DUSPs are not known to directly dephosphorylate MAPKs (Table 2). Besides dephosphorylating MAPKs, DUSPs (such as DUSP6, DUSP8, DUSP14, and DUSP22) also dephosphorylate non-MAPK substrates [28–34]. Collectively, DUSPs can target various substrates and regulate multiple biological pathways.

**Table 1:**
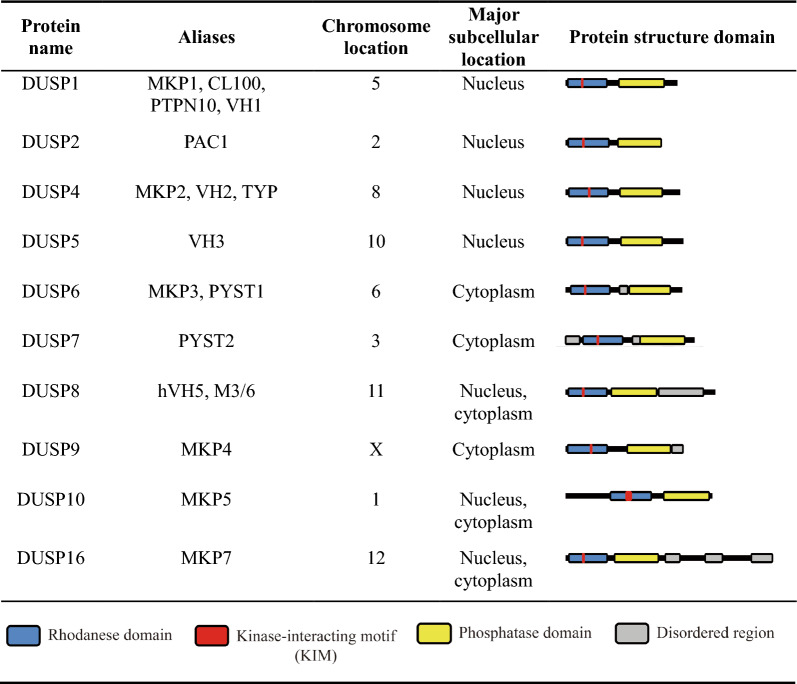
Human typical DUSPs (MKPs) containing KIM

**Table 2:**
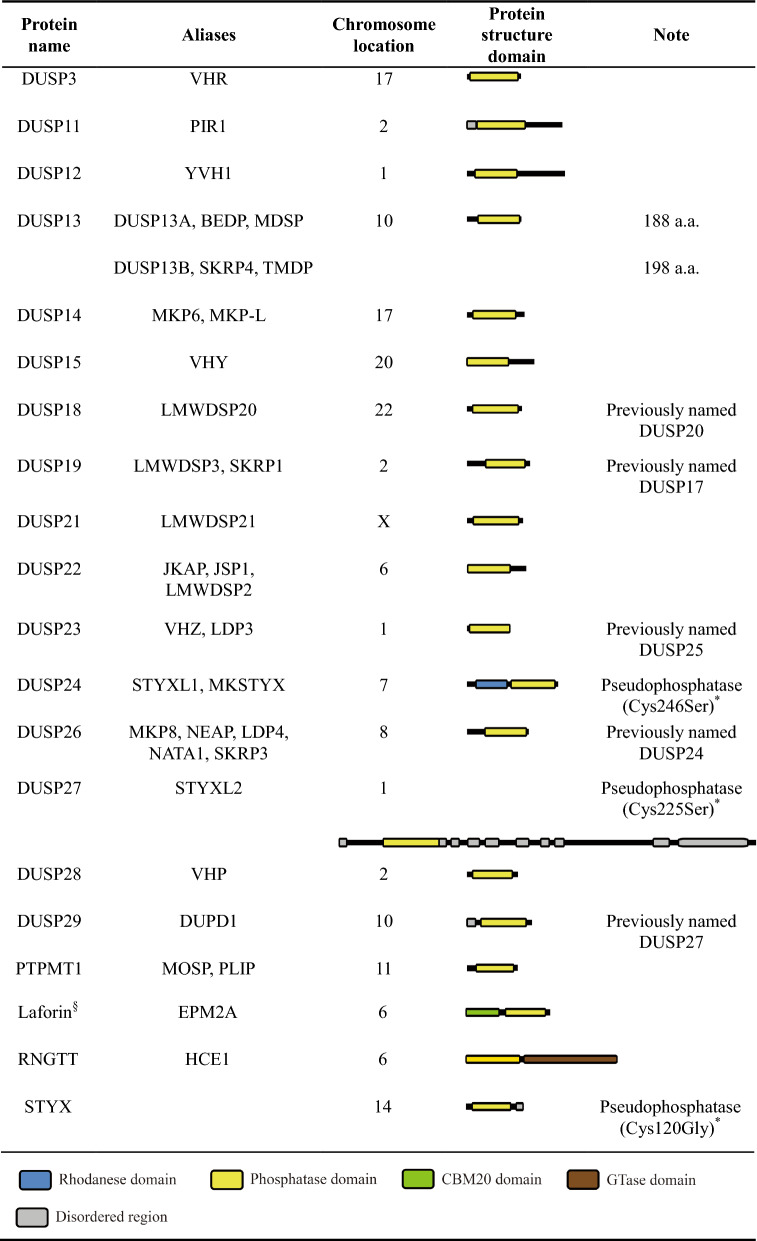
Human atypical DUSPs lacking KIM

*The catalytic cysteine residue of DUSP24 and DUSP27 is mutated into a serine residue, resulting in the loss of phosphatase ability. The catalytic cysteine residue of STYX is mutated into a glycine residue

Laforin^§^ is a glycogen phosphatase

*CBM20* carbohydrate binding type-20

**Fig. 3:**
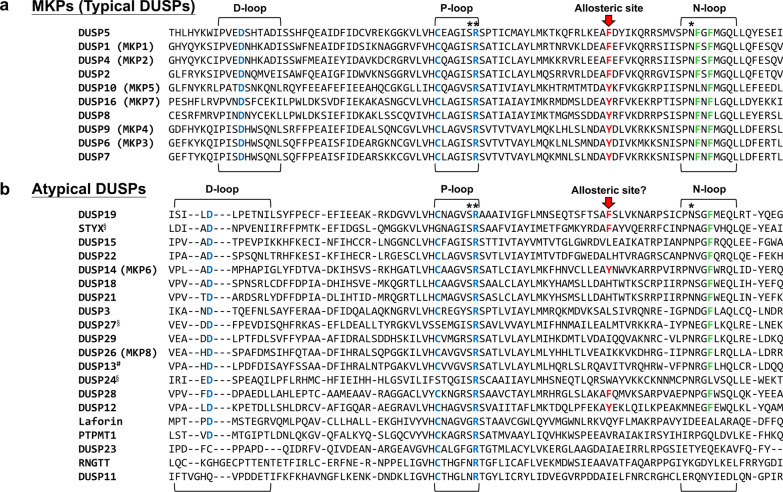
DUSP protein sequence alignments. **a** Protein sequence alignment of 10 MKPs (typical DUSPs). The allosteric sites of DUSP10 (Tyr435) and DUSP16 (Tyr271), as well as the aligned allosteric sites of other MKPs are labeled in *red* and marked by a *red arrow*. The F-X-F motifs are labeled in *green*. **b** Protein sequence alignment of 20 atypical DUSPs. The conserved catalytic residues in the D-loop (except DUSP23, RNGTT, and DUSP11) and P-loop (except the pseudophosphatases DUSP24, DUSP27, and STYX) are labeled in *blue*. A hydrogen bonding network is formed by the conserved N-loop asparagine (except laforin, DUSP23, RNGTT, and DUSP11), P-loop arginine, and P-loop serine residues: the hydrogen bonding network residues are marked by *asterisks*. DUSP24^§^, DUSP27^§^, and STYX^§^ are pseudophosphatases. DUSP13^#^ indicates the DUSP13A protein sequence. The protein sequence alignment was operated by MUSCLE 5

**Fig. 4:**
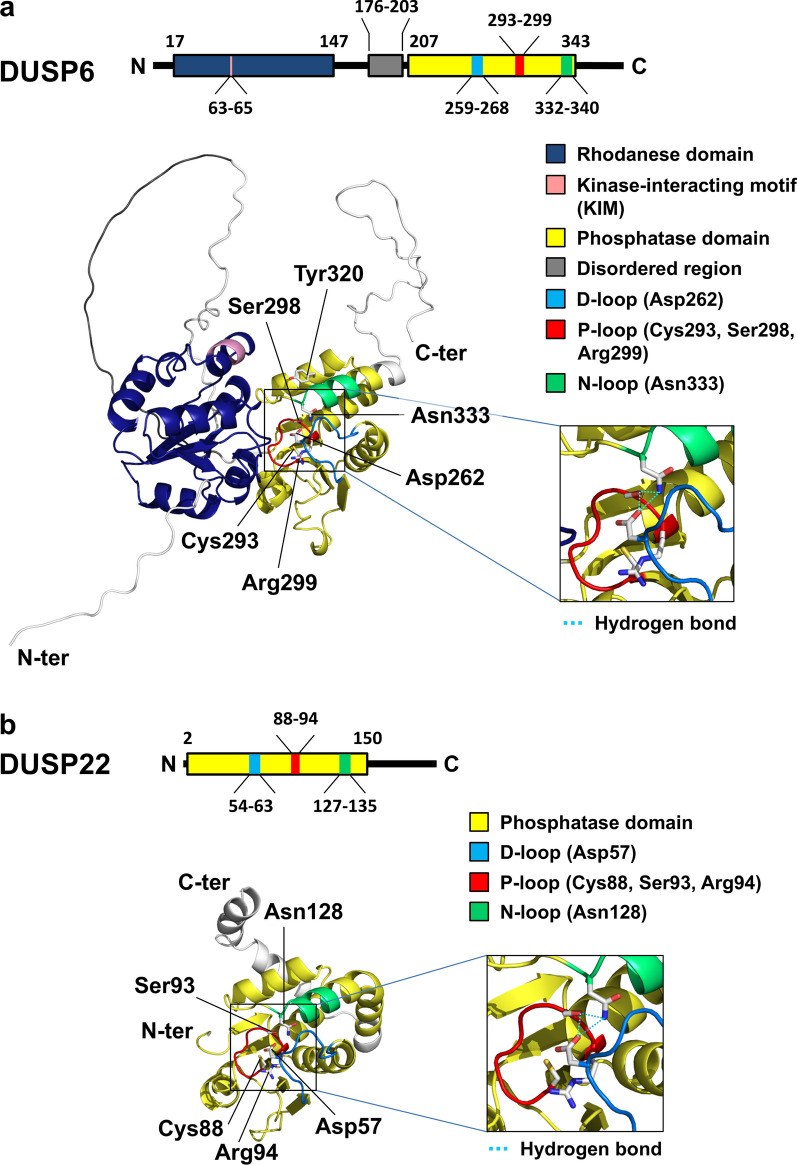
Protein structures of DUSPs. **a** Domain structure of the DUSP6 (MKP3, a typical DUSP). DUSP6 phosphatase catalytic site is comprised by Asp262 (in D-loop), Cys293 (in P-loop), and Arg299 (in P-loop) residues. Asp262 (in D-loop), Ser298 (in P-loop), and Asn333 (in N-loop) form a hydrogen bonding network, maintaining the catalytically favorable conformation of DUSPs. The Tyr320 residue is the conserved allosteric site in DUSP6 based on protein sequence alignment. **b** Domain structure of DUSP22 (an atypical DUSP). DUSP22 phosphatase catalytic site is comprised by Asp57 (in D-loop), Cys88 (in P-loop), and Arg94 (in P-loop) residues. The hydrogen bonding network of DUSP22 is formed by Asp57 (in D-loop), Ser93 (in P-loop), and Asn128 (in N-loop). The catalytic residues are labeled in *red*. The full-length protein structures were generated by AlphaFold v2 and obtained from the AlphaFold Protein Structure Database (DUSP6: AF-Q16828-F1-v6, DUSP22: AF-Q9NRW4-F1-v6). The structure visualization was operated by PyMOL molecular visualization system

**Fig. 5:**
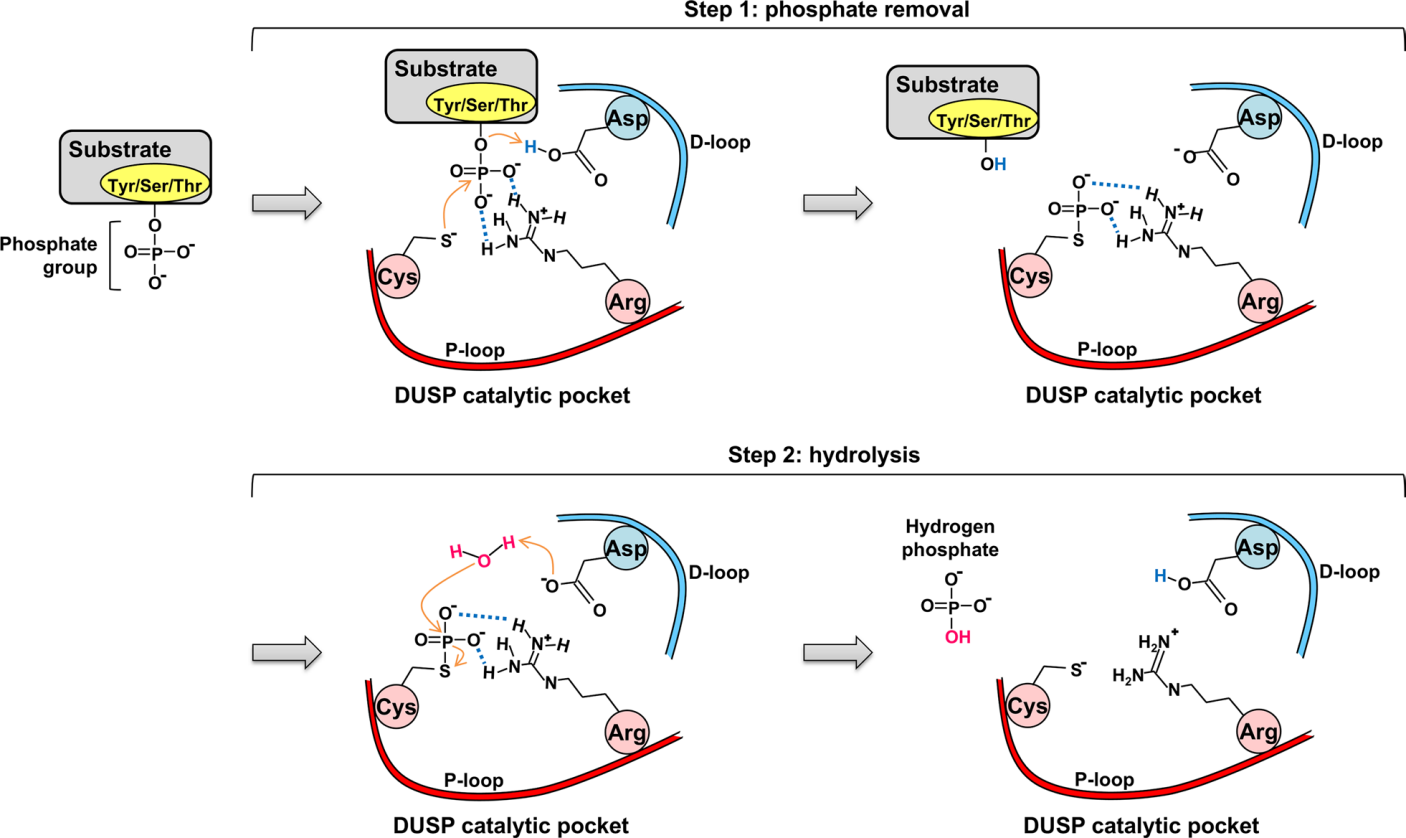
DUSP catalytic mechanism. The dephosphorylation process can be divided into 2 steps, the phosphate removal step and the hydrolysis step. In the phosphate removal step, the P-loop nucleophilic cysteine (unprotonated) breaks the phosphorus-oxygen bond on the phosphate group by attacking the phosphorus atom, forming a cysteinyl-phosphate intermediate. D-loop aspartic acid (protonated) donates a proton to the oxygen atom of the substrate, facilitating the removal of the phosphate group and the release of the dephosphorylated substrate. The P-loop arginine residue stabilizes the substrate phosphate group by forming salt bridge (a combination of hydrogen bonding and ionic bonding) interactions, followed by stabilizing the cysteinyl-phosphate intermediate. In the subsequent hydrolysis step, the unprotonated D-loop aspartate activates a water molecule and abstracts a proton from the water molecule. The activated water molecule facilitates the hydrolysis of cysteinyl-phosphate intermediate, resulting in the release of a hydrogen phosphate, and the DUSP catalytic pocket returns to its original state

MKPs (typical DUSPs) and atypical DUSPs contain a phosphatase domain with 3 conserved loops: D-loop, phosphate-binding loop (P-loop), and N-loop (Fig. 3). The N-loop cooperates with the P-loop and the D-loop to form a hydrogen bonding network (Fig. 4), which maintains DUSP catalytic pocket in a catalytically favorable conformation [2]. DUSP catalytic pocket contains 3 conserved catalytic residues, aspartic acid in the D-loop (except DUSP23, RNGTT, and DUSP11), cysteine in the P-loop (except the pseudophosphatases DUSP24, DUSP27, and STYX), and arginine in the P-loop [35, 36] (Fig. 3). The dephosphorylation process can be divided into 2 steps: the phosphate removal step and the hydrolysis step [2, 6, 37, 38] (Fig. 5). In the phosphate removal step (step 1), the P-loop nucleophilic cysteine (unprotonated) breaks the phosphorus-oxygen bond on the phosphate group by attacking the phosphorus atom, forming a cysteinyl-phosphate intermediate. At the same time, D-loop aspartic acid (protonated) donates a proton to the oxygen atom of the substrate, facilitating the removal of the phosphate group and the release of the dephosphorylated substrate. In addition, the P-loop arginine residue stabilizes the substrate phosphate group by forming salt bridge (a combination of hydrogen bonding and ionic bonding) interactions, followed by stabilizing the cysteinyl-phosphate intermediate. In the subsequent hydrolysis step (step 2), the unprotonated D-loop aspartate activates a water molecule and abstracts a proton from the water molecule. The activated water molecule facilitates the hydrolysis of cysteinyl-phosphate intermediate, resulting in the release of a hydrogen phosphate, and the DUSP catalytic pocket returns to its original state. In vitro binding [13] and crystal structure analyses [13, 39–43] suggest that most of DUSPs (except DUSP2, DUSP6, DUSP7, DUSP9, DUSP24, and DSUP29) contain an adjacent pocket, which cooperates with the catalytic pocket to interact with double-phosphorylated T-X-Y motifs of MAPKs. Specifically, the catalytic and adjacent pockets of DUSP3 concurrently bind to threonine and tyrosine residues, respectively, within a 11-a.a. double-phosphorylated substrate peptide of p38 [13]. It is possible that DUSPs also can concurrently bind to double-phosphorylated residues of non-MAPK substrates. In addition, an F-X-F motif in the N-loop of DUSP16 directly interacts with JNK and is crucial for dephosphorylation of JNK [44]. Besides the catalytic residues (aspartic acid, cysteine, and arginine), a conserved tyrosine residue (e.g. DUSP10 Tyr435 and DUSP16 Tyr271) [45, 46] or phenylalanine residue in the phosphatase domain of MKPs (typical DUSPs) is an allosteric site critical for catalytic activity and MAPK binding (Fig. 3). Interestingly, alignment results show that the allosteric site tyrosine or phenylalanine residue, as well as the F-X-F motif found in MKPs (typical DUSPs) is not conserved in atypical DUSPs. It would be interesting to study whether the aligned residues/motifs in atypical DUSPs are potential allosteric sites or F-X-F like motifs.

DUSP expression and activation are regulated by gene variants [47], transcription regulation [48–51], and post-translational modification [36]. DUSPs are involved in the pathogenesis of various human inflammatory and chronic diseases, including autoimmune diseases (Table 3), allergic diseases (Table 4), inflammatory bowel diseases (IBDs) (Table 5), metabolic diseases (Table 6), cardiovascular diseases (Table 7), and cancers. In this review, we present a comprehensive review of the roles of DUSPs, including MKPs (typical DUSPs) and atypical DUSPs, in human inflammatory and chronic diseases.

**Table 3:** DUSPs in autoimmune diseases

DUSP	Diseases	Expression in patients or animal models	Description (function)	Reference
DUSP1	Multiple sclerosis	Decrease	DUSP1 protein and mRNA levels are decreased in the white matter tissue of brain from multiple sclerosis patients	[58]
	RA	Decrease	DUSP1 deficiency enhances symptoms in arthritis mice	[56, 57, 59]
	Psoriasis	Decrease	DUSP1 mRNA levels are decreased in the skin of psoriasis patients	[62, 63]
	Glomerulonephritis	Decrease	DUSP1 mRNA levels are decreased in the tubulointerstitial of glomerulonephritis patients	[64]
DUSP2	LN	Decrease	DUSP2 protein levels are decreased in the kidney of LN patients DUSP2 overexpression mitigates nephritis in MRL/lpr LN mice DUSP2 inhibits Th17 differentiation by dephosphorylating STAT3	[33, 66]
	RA	Increase	DUSP2 mRNA levels are induced in activated eosinophil/lymphocyte cell lines DUSP2 KO mice are resistant to arthritis induction	[67]
DUSP3	RA	Decrease	miR-1246 decreases DUSP3 mRNA levels and increases IL-6 mRNA levels in THP-1 macrophages miR-1246 levels are increased in the plasma of RA patients	[71]
DUSP4	SLE	Increase	DUSP4 mRNA levels are increased in the T cells of juvenile-onset SLE patients DUSP4 levels are positively correlated with CREMα levels in human Th1/Th17 cells	[48]
	AS	Increase	DUSP4 mRNA levels are increased in the T cells of AS patients	[77]
DUSP5	RA	Decrease	Th17 population and STAT3 activation are increased by DUSP5 deficiency in CIA mice DUSP5 overexpression of attenuates the induction of arthritis in mice	[80]
	AS	Increase	DUSP5 mRNA levels are increased in the T cells of AS patients	[77]
DUSP6	AS	Increase	DUSP6 levels are correlated with TNF-α levels in AS patients	[77]
	Autoimmune arthritis	Increase	DUSP6 KO mice are resistant to arthritis induction	[83, 84, 87]
DUSP7	AS	Increase	DUSP7 mRNA levels are induced in the T cells of AS patients	[77]
	RA	Decrease	DUSP7 mRNA levels are decreased in the T cells of RA patients	[91]
DUSP11	RA	Decrease	Serum anti-DUSP11 autoantibody is increased and correlated with disease activity in RA patients	[96]
DUSP12	MAS	SNP	One DUSP12 variant is increased in MAS patients One DUSP12 variant causes P81R mutation at DUSP12 phosphatase domain	[47]
DUSP14	AS	Increase	DUSP14 mRNA levels are increased in the T cells of AS patients and correlated with TNF-α levels	[77]
	Axial spondyloarthritis/AS	Decrease	DUSP14 mRNA levels are decreased in the blood of axial spondyloarthritis/AS patients DUSP14 inactivates TAK1, JNK, and IKK by dephosphorylating TAB1	[29, 101]
DUSP16	Autoimmune inflammation	Increase	DUSP16-deficient mice display reduced EAE induction, increased IL-2 production, and reduced Th17 differentiation in CD4^+^ T cells Th17 proliferation is enhanced by DUSP16 deficiency in murine dendritic cells	[106]
	Autoimmune inflammation	Decrease	miR-338-3p decreases DUSP16 mRNA levels in murine dendritic cells	[107]
DUSP22	AS	Decrease	DUSP22 mRNA levels are decreased in the T cells of AS patients and correlated with disease activity DUSP22 KO mice display AS-like symptoms	[77]
	SLE	Decrease	DUSP22 protein levels are decreased in the T cells of SLE patients and correlated with nephritis DUSP22 deficiency causes multi-organ inflammation and autoantibody induction in mice DUSP22 dephosphorylates Lck at Tyr394 and UBR2 at Ser1694/Tyr1697, inactivating Lck	[30, 31, 77, 110]
	RA	Decrease	Serum DUSP22 levels are decreased in RA patients and correlated with therapy sensitivity	[111, 112]
	SS	Unknown	Methylation of the DUSP22 promoter is enhanced in SS patients	[115]
DUSP23	SLE	Increase	DUSP23 mRNA levels are increased in the CD4^+^ T cells of SLE patients and correlated with disease severity	[119]

**Table 4:** DUSPs in allergic diseases

DUSP	Diseases	Expression in patients or animal models	Description (function)	Reference
DUSP1	Allergic rhinitis/asthma	Decrease	DUSP1 mRNA levels are decreased in the bronchial epithelial cells of allergic rhinitis/asthma patients	[122, 123]
DUSP2	Th2-mediated allergy	Decrease	DUSP2 mRNA levels are decreased in Th2 cells in androgen receptor deficient mice DUSP2 suppresses Th2-mediated allergic inflammation	[50]
DUSP8	Allergic asthma	Increase	DUSP8 protein levels are increased in the T cells of asthma patients DUSP8 dephosphorylates Pur-α, leading to Pur-α cytoplasm translocation and IL-9 reduction	[32]
DUSP14	Allergic asthma	Decrease	DUSP14 protein levels are decreased in the lung of asthma mice	[131]

**Table 5:** DUSPs in IBDs

DUSP	Diseases	Expression in patients or animal models	Description (Function)	Reference
DUSP2	UC	Decrease	DUSP2 mRNA levels are decreased in the PBMCs of UC patients Methylation frequency of the DUSP2 promoter is increased in UC patients DUSP2 deficiency exacerbates symptoms in colitis mice DUSP2 dephosphorylates and inactivates STAT3	[33]
DUSP6	IBD	Decrease	DUSP6 KO mice exacerbate colitis in enterocolitis murine model DUSP6 deficiency promotes Th1 differentiation in murine primary CD4^+^ T cells	[134]
	UC	Increase	DUSP6 mRNA levels are increased in the inflamed colonic mucosa and epithelial cells of UC patients DUSP6 deficiency reduces symptoms in colitis mice	[135, 137]
DUSP11	UC/CD	Decrease	DUSP11 mRNA levels are decreased in the sigmoid colon or the terminal ileum of UC and CD patients	[138]
DUSP16	IBD	Unknown	DUSP16 is associated with IBD causal factor KSR1	[139]
DUSP22	UC/CD	Decrease	DUSP22 protein levels are decreased in the intestine mucosa of active IBD patients and correlated with disease activity	[141]
DUSP28	UC	Decrease	DUSP28 mRNA levels are negatively correlated with UC	[142]

**Table 6:** DUSPs in obesity, diabetes, and other metabolic diseases

DUSP	Diseases	Expression in patients or animal models	Description (function)	Reference
DUSP1	Diabetic nephropathy	Decrease	DUSP1 mRNA levels are decreased in the kidney of diabetic nephropathy patients DUSP1 protein levels are decreased in the kidney of diabetic nephropathy rats DUSP1 overexpression ameliorates diabetic nephropathy in diabetic mice	[146, 147]
	Obesity	Increase	DUSP1 protein levels are increased in PBMCs/subcutaneous adipose tissue of human obese participants and the liver of obese mice DUSP1 deficiency enhances PPAR-α activity in murine hepatocytes DUSP1 KO mice are resistant to diet-induced obesity	[148–150]
	NAFLD	Increase	DUSP1 mRNA levels are increased in the liver of obese NASH patients DUSP1 deficiency reduces disease symptoms in NASH mice DUSP1 promotes hepatocyte apoptosis and autophagy by inactivating AMPK	[149, 152]
DUSP3	NAFLD/Obesity	Decrease	DUSP3 mRNA levels are decreased in the liver of obese mice DUSP3-deficient mice display increased body weight and NAFLD DUSP3 deficiency enhances hepatocellular carcinoma in obese mice	[153]
DUSP4	NAFLD/Obesity/Diabetes	Increase	DUSP4 protein levels are increased in the liver of NASH patients and mice DUSP4 deficiency reduces insulin resistance and hepatic steatosis in obese mice DUSP4 deficiency enhances IGF1-AKT pathway in mice	[73]
DUSP5	Diabetic cardiomyopathy	Decrease	DUSP5 protein levels are decreased in the heart of diabetic cardiomyopathy mice	[156, 157]
DUSP6	NAFLD/Obesity/Diabetes	Increase	DUSP6 protein levels are increased in the liver of obese mice DUSP6 deficient mice are resistant to diet-induced obesity DUSP6 deficiency reduces glycemia and NAFLD in mice DUSP6 enhances PEPCK and G6Pase transcription by promoting FOXO1 nuclear translocation	[34, 163–165]
	Diabetic cardiovascular disease	Decrease	DUSP6 protein levels are decreased in human and murine aortic endothelial cells DUSP6 overexpression decreases monocyte adhesion in human aortic endothelial cells	[169]
DUSP7	NAFLD/Obesity	Decrease	DUSP7 protein levels are decreased in the liver of NAFLD patients and correlated with disease activity DUSP7 deficiency increases insulin resistance and NAFLD in obese mice	[172]
DUSP8	Diabetes	Increase (feedback regulation)	DUSP8 deficiency causes glucose intolerance and insulin resistance in mice DUSP8 mRNA levels are increased in the hypothalamus of T2D patients and obese/diabetic mice as feedback regulation	[175]
	Diabetic cardiomyopathy	Decrease	miR-21 downregulates DUSP8 protein levels, enhancing collagen synthesis in rat cardiac fibroblasts	[176]
DUSP9	Diabetes/Obesity	Increase (feedback regulation)	DUSP9 protein levels are increased in the adipose tissue of obese/diabetic mice DUSP9 overexpression improves glucose tolerance and inhibits adipocyte differentiation as feedback regulation	[179, 182]
	GDM	Increase	DUSP9 protein levels are increased in the umbilical cord blood and placenta of GDM patients and correlated with disease symptoms DUSP9 suppresses PI3K and AKT activation by dephosphorylating IRS-1	[184]
	NAFLD/Obesity	Decrease	DUSP9 protein levels are decreased in the liver of obese mice DUSP9 deficiency enhances NAFLD symptoms in obese mice DUSP9 inhibits ASK1-p38/JNK signaling in murine hepatocytes	[185]
DUSP10	NAFLD/Obesity	Decrease	DUSP10 protein levels are decreased in the liver tissue of NAFLD mice DUSP10 deficiency enhances NAFLD in obese mice DUSP10 suppresses CIDEA/CIDEC transcription by inhibiting p38, AFT2, and PPAR-γ activation DUSP10 downregulation is mediated by TRIM7-induced K63-linked ubiquitination at Lys452 residue	[188–190]
DUSP12	Diabetes/Diabetic cardiomyopathy	Increase (feedback regulation)	DUSP12 deficiency suppresses IRS1-AKT-GLUT4-mediated insulin signaling DUSP12 deficiency causes cardiomyopathy and cardiac dysfunction in obese mice DUSP12 inactivates JNK and p38 by dephosphorylating ASK1 DUSP12 protein levels are increased in the heart tissue of obese mice as feedback regulation	[195]
	NAFLD/Obesity	Decrease	DUSP12 protein levels are decreased in the liver tissue of obese mice DUSP12 deficiency enhances insulin resistance and NAFLD in obese mice	[196]
DUSP14	β-cell proliferation	Increase (feedback regulation)	DUSP14 deficiency increases islet β-cell proliferation in mice Enhanced murine β-cells proliferation increases DUSP14 protein levels as feedback regulation	[197]
	NAFLD/Obesity/Diabetes	Decrease	DUSP14 protein levels are decreased in the liver of obese mice and NAFLD patients DUSP14 overexpression reduces insulin resistance but induces glycogenesis in obese mice DUSP14 overexpression enhances IRS1-AKT signaling	[199]
DUSP15	Diabetic cardiomyopathy	Decrease	DUSP15 mRNA and protein levels are decreased in the heart tissue of diabetic cardiomyopathy mice	[201]
DUSP16	NAFLD/Obesity/Diabetes	Decrease	DUSP16 mRNA levels are decreased in the liver of obese mice DUSP16 deficiency enhances hepatic lipid synthesis, hepatic inflammation, and insulin resistance in obese mice	[204]
DUSP22	Diabetic nephropathy	Decrease	DUSP22 protein levels are decreased in high glucose-treated murine mesangial cells DUSP22 overexpression decreases cell proliferation, cell fibrosis, and inflammation in high-glucose-treated murine mesangial cells	[205]
	NAFLD/Obesity	Decrease	DUSP22 protein levels are decreased in the liver of NASH patients and correlated with disease severity, enhancing hepatocellular carcinoma progression DUSP22 deficiency enhances FAK activation and NF-κB/ERK signaling in obese mice	[207]
DUSP26	Diabetes	Increase	DUSP26 overexpression increases rat INS-1 β cell apoptosis DUSP26 mRNA levels are decreased in pancreatic islet after inducing insulin secretion in diabetic mice	[211]
	Diabetic cardiomyopathy	Decrease	DUSP26 protein levels are decreased in the heart of diabetic mice DUSP26 overexpression reduces myocardial fibrosis/hypertrophy in diabetic mice	[213]
	Diabetic nephropathy	Decrease	DUSP26 protein levels are decreased in the kidney of diabetic nephropathy patients DUSP26 deficiency enhances ROS production, MAP kinas activation, and nephropathy development in diabetic mice	[214]
	Acute kidney injury	Decrease	DUSP26 protein levels are decreased in acute kidney injury patients Methylation frequencies of the DUSP26 promoter regions are increased in the kidney tissue of acute kidney injury mice DUSP26 inhibits apoptosis by dephosphorylating p53 at Ser312 residue	[215]
	NAFLD/Obesity	Decrease	DUSP26 protein levels are decreased in the liver of obese mice DUSP26 deficiency enhances hepatic lipid accumulation, insulin resistance, and liver damage in obese mice	[216]

**Table 7:** DUSPs in cardiovascular diseases

DUSP	Diseases	Expression in patients or animal models	Description (Function)	Reference
DUSP1/4	Cardiac hypertrophy/ Heart failure	Increase (feedback regulation)	DUSP1/DUSP4 dKO mice spontaneously develop cardiomyopathy Cardiac hypertrophy mice and heart failure patients display an increase of DUSP1/DUSP4 protein levels in the heart tissue as feedback response	[220–222]
DUSP3	AMI	Increase	DUSP3 protein levels are increased in the heart of AMI mice DUSP3 deficiency alleviates disease symptom in AMI mice	[223]
DUSP6	MI	Increase	DUSP6 mRNA and protein levels are increased in the neutrophils, macrophages, and heart of MI rats DUSP6 deficiency reduces disease symptoms in MI rats	[51, 224, 225]
DUSP7	Dilated cardiomyopathy	Increase	DUSP7 overexpression promotes dilated cardiomyopathy in mice	[228]
DUSP8	Dilated cardiomyopathy/ Cardiac hypertrophy	Increase	DUSP8 mRNA levels are increased in the heart of dilated cardiomyopathy mice and patients DUSP8 overexpression promotes cardiac hypertrophy in mice	[229, 230]
DUSP12	Dilated cardiomyopathy/ Cardiac hypertrophy	Decrease	DUS12 protein levels are decreased in the heart of cardiac hypertrophy mice and cardiomyopathy patients DUSP12 deficiency enhances JNK activation in cardiac hypertrophy mice	[231]
	Myocardial I/R injury	Decrease	DUSP12 protein levels are decreased in the heart of myocardial I/R rat DUSP12 overexpression suppresses MI in I/R rats	[232]
	Hypertension	Increase	DUSP12 mRNA levels are increased in hypertension and left ventricular remodeling patients	[234]
DUSP14	Dilated cardiomyopathy	Decrease	DUSP14 protein levels are decreased in the heart of dilated cardiomyopathy patients with heart failure DUSP14 deficiency induces TAK1, JNK, and p38 activation and cardiac hypertrophy in mice	[235]
	Myocardial I/R injury	Decrease	DUSP14 levels are decreased in the heart of myocardial I/R injured mice DUSP14 deficiency enhances inflammation in I/R-injured mice DUSP14 downregulation by miR-217 increases MAPK/NF-κB activation in rat cardiac myoblasts DUSP14 downregulation induced by miRNA-18a-5p is reversed by circAPBB2 overexpression	[236–239]
DUSP16	Atherosclerosis	Increase	DUSP16 protein levels are increased in the HUVECs by monocyte adhesion induction DUSP16 deficiency decreases IRF-1 and VCAM-1 protein levels	[240]
DUSP22	CHD	Decrease	Serum DUSP22 levels are decreased in CHD patients and correlated with coronary artery stenosis and Th1/Th17 populations	[242]
DUSP26	Cardiac hypertrophy	Increase (feedback regulation)	DUSP26 deficiency enhances TAK1 activation and disease symptoms after cardiac hypertrophy induction DUSP26 protein levels are increased in the heart of cardiac hypertrophy mice as a feedback regulation	[246]

## The roles of DUSPs in cell signaling of autoimmune diseases

Autoimmune diseases are chronic and incurable diseases. Autoimmune diseases are caused by dysregulation of B cells, T cells, and macrophages, leading to multiple organ damages by hyperactivation of inflammatory responses [52, 53]. About 150 different types of autoimmune diseases have been identified, and these diseases affect around 3–5 % of world’s population [53]. DUSPs (including DUSP1, DUSP2, DUSP3, DUSP4, DUSP5, DUSP6, DUSP7, DUSP11, DUSP12, DUSP14, DUSP16, DUSP22, and DUSP23) play important roles in the pathogenesis of autoimmune diseases, such as rheumatoid arthritis (RA), systemic lupus erythematosus (SLE), and ankylosing spondylitis (AS) (Table 3). Studying and understanding of the DUSPs-mediated regulatory mechanisms of these diseases will help develop better diagnostic and therapeutic strategies.

### DUSP1 (MKP1, PTPN10)

DUSP1 (also named MAPK phosphatase 1, MKP1 or protein tyrosine phosphatase non-receptor type 10, PTPN10) functions as a negative regulator of MAPKs by dephosphorylating ERK, JNK, and p38 [36, 54, 55]. DUSP1 knockout (KO) mice show an increase of the proinflammatory cytokines IL-10 and TNF-α upon lipopolysaccharide (LPS) challenge compared to wild-type mice [56]. The increased levels of IL-10 and TNF-α are suppressed by p38 inhibitor in LPS-stimulated macrophages of DUSP1 KO mice. The exacerbated symptoms of experimental autoimmune encephalomyelitis (EAE) model using DUSP1-deficient bone marrow chimeric mice [57], suggesting that DUSP1 downregulation may be involved in autoimmune responses. Consistently, DUSP1 mRNA and protein levels are decreased in the white matter tissue of brain from patients with progressive multiple sclerosis compared to those of non-multiple sclerosis individuals [58]. In addition, DUSP1 KO mice display accelerated arthritis symptoms during the induction of collagen-induced arthritis (CIA) [56, 59]. The severe arthritis in DUSP1 KO mice may be due to the enhancement of osteoclast differentiation [59]. The proinflammatory stimuli (e.g., IL-1β and TNF-α) induce DUSP1 mRNA and protein levels in primary fibroblast-like synoviocytes of RA patients, resulting in the suppression of ERK, JNK, and p38 activation [60]. The data suggest that DUSP1 attenuates the pathogenesis of RA by inhibiting the activation of macrophage, osteoclast, and synoviocytes.

DUSP1 mRNA and protein levels are decreased in LPS-stimulated human HaCaT keratinocytes [61]. Consistently, DUSP1 mRNA levels are decreased in the mesenchymal stem cells and the skin tissue of psoriasis patients [62, 63]. DUSP1 overexpression inactivates the ERK-Elk1-Egr1 pathway in HaCaT keratinocytes upon stimulation of the proliferation factors, resulting in apoptosis of keratinocytes [62]. Similarly, transcriptomic analysis of glomerulonephritis patients shows that DUSP1 mRNA levels are decreased in the tubulointerstitial tissue of patients compared to healthy controls [64]. Consistently, immunohistochemical staining shows that the phosphorylation levels of both ERK and p38 are increased in the tubulointerstitial tissue of glomerulonephritis patients. Overexpression of DUSP1 reduces TNF-α-induced activation of p38, ERK, and JNK in human primary tubular epithelial cells [64]. These results suggest that DUSP1 downregulation contributes to the pathogenesis of psoriasis and glomerulonephritis.

### DUSP2 (PAC1)

DUSP2 (also named Phosphatase of activated cells 1, PAC1) is a typical DUSP that dephosphorylates ERK and p38 in vitro [65]. DUSP2 also dephosphorylates the transcription factor STAT3 at Tyr705 and Ser727 residues, resulting in the inactivation of STAT3 and subsequent inhibition of Th17 differentiation [33]. DUSP2 mRNA and protein levels are decreased in the kidney tissue of lupus nephritis (LN) patients compared to those of healthy individuals [66]. Consistently, overexpression of DUSP2 by adeno-associated virus (AAV) transduction in MRL/lpr LN mice mitigates nephritis symptoms including proteinuria, blood urea nitrogen (BUN), and kidney pathological features. DUSP2 overexpression decreases proinflammatory cytokines TNF-α, IL-6, and IL-1β levels in the serum and kidney tissue of MRL/lpr LN mice, reducing STAT3 activation and COX2 expression in the kidney tissue [66]. These findings suggest that DUSP2 prevents autoimmune LN, possibly via its inhibition of STAT3 activation.

Interestingly, DUSP2 mRNA levels are greatly increased in mast cells derived from cord blood and activated human B cell, T cell, mast cell, and eosinophil cell lines [67]. Unlike the inactivation of ERK/p38 by DUSP2 in vitro [65], ERK and p38 activation is decreased by DUSP2 KO in activated murine macrophages and mast cells [67]. The mRNA levels of IL-6 and TNF-α, as well as the production of IL-2 are decreased by DUSP2 KO in murine macrophages upon LPS stimulation in vitro. Furthermore, DUSP2 KO mice are resistant to arthritogenic serum K/BxN-induced arthritis compared to wild-type mice [67]. These results suggest that DUSP2 may promote macrophages/mast cell-mediated arthritis.

### DUSP3 (VHR)

DUSP3 (also named Vaccinia H1-related phosphatase, VHR) is an atypical DUSP, which does not contain KIM but still shows phosphatase activity against MAPKs [68, 69]. DUSP3 specifically dephosphorylates the MAPKs ERK and JNK, but not p38 in T cells under TCR signaling [24, 25]. Moreover, DUSP3 inhibits TCR-induced NFAT and AP-1 activation in Jurkat T cells [24]. Besides T cells, DUSP3-deficient mice show the increase of M2 macrophages and resistance to LPS-induced septic shock [70]. These findings suggest that DUSP3 contributes to T-cell- and macrophage-mediated immune responses. Interestingly, RA patients display an increase of high-density lipoprotein (HDL) carrying miR-1246, which targets the 3′-untranslated region (3′-UTR) of DUSP3 [71]. The plasma levels of HDL-miR-1246 are higher in active RA patients than RA remission patients. Moreover, HDLs isolated from RA patients trigger downregulation of DUSP3 mRNA levels and upregulation of IL-6 mRNA levels in human THP-1 macrophages [71]. The data suggest that DUSP3 downregulation may contribute to macrophage-mediated inflammation of RA patients.

### DUSP4 (MKP2)

DUSP4 (also named MAPK phosphatase 2, MKP2) is categorized as a typical DUSP and is predominantly expressed in the nucleus [36]. DUSP4 dephosphorylates the MAPKs ERK and p38 in vitro [72]. The activations of p38 and JNK are induced in murine liver tissue [73] and LPS-stimulated primary macrophages of DUSP4 KO mice [74]. DUSP4 deficiency in murine macrophages results in ERK hyperactivation and proinflammatory cytokine overproduction, leading to the excessive progression of *Pseudomonas aeruginosa*-induced bacterial pneumonia [75]. There is a negative feedback loop between DUSP4 and ERK. ERK phosphorylates and induces stabilization and activation of DUSP4; the activated DUSP4 then reciprocally dephosphorylates and inhibits ERK [36]. DUSP4 also inhibits IL-2 production and T-cell proliferation through STAT5 [76]. Overexpression of DUSP4 results in a decrease of IL-2 mRNA levels in human T cells [48]. Conversely, DUSP4-deficient mice show an enhancement of STAT5 phosphorylation and IL-2 production in T cells, resulting in CD4^+^ T cell hyperproliferation [76].

DUSP4 mRNA levels are increased in the peripheral blood T cells of patients with AS [77] or juvenile-onset SLE [48] than those of healthy individuals. DUSP4 mRNA levels are positively correlated with the mRNA levels of the transcription factor CREMα in human Th1 and Th17 cells [48, 78]. The mRNA levels of DUSP4 are decreased in CREM-deficient Jurkat T cells. Moreover, chromatin immunoprecipitation assay data indicate that CREMα and the histone acetyltransferase p300 bind to the DUSP4 promoter in Jurkat T cells [48]. The data suggest that CREMα recruits p300 to the promoter region of DUSP4, inducing DUSP4 transcription in SLE T cells.

### DUSP5 (VH3)

DUSP5 (also named VH1-like phosphatase-3, VH3) protein is phosphorylated by the kinase ERK at Thr321, Ser346, and Ser376 residues in Cos-1 cells; among the three ERK-mediated phosphorylation residues, Thr321 phosphorylation maintains DUSP5 protein stabilization [36, 79]. The phosphorylated and stabilized DUSP5 then reciprocally dephosphorylates and inactivates ERK [36, 79]. Overexpression of DUSP5 in mice by electroporation attenuates the CIA induction [80]. The protein levels of IL-1β, IL-6, TNF-α, ERK, and STAT3 are decreased by DUSP5 overexpression in ankle joint tissue of symptomatic mice. Moreover, the percentage of Th17 cells is decreased, and the percentage of Treg is increased in the spleen of DUSP5-overexpressing mice during CIA induction. Conversely, the Th17 population and STAT3 activation in the spleen of symptomatic mice are increased by DUSP5 siRNA knockdown [80]. In addition, DUSP5 mRNA levels are increased in peripheral blood T cells of AS patients [77]. These findings suggest that DUSP5 inactivates STAT3 and inhibits Th17 population, resulting in the suppression of autoimmune diseases, such as RA.

### DUSP6 (MKP3)

DUSP6 (also named MAPK phosphatase 3, MKP3) dephosphorylates and inactivates ERK. ERK reciprocally phosphorylates DUSP6 at Ser159 and Ser197 residues, leading to proteasomal degradation of DUSP6 protein [81, 82]. DUSP6 transcription is promoted by the ERK-responsive transcription factor Ets1/2 in FGF signaling [49]. DUSP6 is also involved in the suppression of murine follicular helper T (T_FH_) cell proliferation [83]. DUSP6 mRNA levels are positively correlated with TNF-α mRNA levels in AS patients [77]. Besides AS, DUSP6 is involved in the induction of autoimmune arthritis murine model [84]. The K/BxN TCR transgenic mice spontaneously develop inflammatory arthritis with high levels of anti-glucose-6-phosphate isomerase autoantibody, which induces arthritis in wild-type mice [85, 86]. DUSP6 KO mice show a resistance to arthritis induced by serum transferring from K/BxN mice [84, 87]. Moreover, symptomatic DUSP6 KO mice show an increase of serum IL-10 levels and IL-10-producing T and B cells. The resistance to arthritis in DUSP6 KO mice is abolished by DUSP6/IL-10 double KO (dKO), suggesting that the protection by DUSP6 deficiency is due to IL-10 signaling [84]. In summary, DUSP6 may contribute to the pathogenesis of arthritis by inhibiting IL-10 production in mice. It would be interesting to study whether this mechanism is applicable to RA or AS patients.

### DUSP7 (PYST2)

DUSP7 (also named PYST2) dephosphorylates and inactivates ERK2 [88, 89]. DUSP7 overexpression leads to ERK dephosphorylation and chromosome misalignment [89]. In contrast, DUSP7 knockdown results in prolonged mitosis [89]. In addition, DUSP7 dephosphorylates and inactivates conventional protein kinase C isoforms, preventing meiotic resumption [90]. DUSP7 mRNA levels are decreased in peripheral blood T cells of RA patients [91]; in contrast, DUSP7 mRNA levels are increased in peripheral blood T cells of AS patients [77]. These findings implicate that DUSP7 may be involved in autoimmune diseases; however, the roles of DUSP7 and chromosome dysregulation in the pathogenesis of autoimmune diseases remain to be clarified.

### DUSP11 (PIR1)

DUSP11 (also named Phosphatase that interacts with RNA/RNP complex 1, PIR1) shows a high affinity to RNAs and proteins [92, 93]. DUSP11 dephosphorylates miRNAs at 5′-triphosphate and enhances miRNA stability [93, 94]. DUSP11 directly dephosphorylates and inhibits TGF-β-activated kinase 1 (TAK1) in macrophages upon LPS stimulation, leading to the suppression of proinflammatory cytokine production [95]. DUSP11-deficient mice show an increase of serum proinflammatory cytokine levels and an enhancement of LPS-induced endotoxic shock. These findings suggest that DUSP11 suppresses macrophage-mediated inflammation by inhibiting TAK1. Notably, serum levels of anti-DUSP11 and anti-PTX3 autoantibodies are increased in RA patients compared to either healthy individuals or patients with other autoimmune diseases [96]. Moreover, the induction of both autoantibodies is positively correlated with the disease activity of RA patients. Thus, anti-DUSP11 and anti-PTX3 autoantibodies are potential diagnostic biomarkers for human RA. It is unclear whether anti-DUSP11 autoantibody promoting RA is somehow involved in blocking the suppressive function of DUSP11 in macrophage-mediated inflammation.

### DUSP12 (YVH1)

DUSP12 (also named Yeast VH1-related phosphatase, YVH1) is an atypical DUSP. DUSP12 plays an important role in promoting cell cycle progression and preventing cell apoptosis [97, 98]. Overexpression of DUSP12 increases G_2_/M cell population, whereas knockdown of DUSP12 increases G_0_/G_1_ cell population and cell senescence [97]. Whole exome sequencing analysis using DNA samples from 8 multiple autoimmune syndrome (MAS) patients, 4 Sjögren's syndrome (SS) patients, and 38 healthy individuals show that one DUSP12 variant is induced in two MAS patients but not in healthy individuals [47]. Moreover, these two MAS patients manifest RA and SS phenotypes regardless of harboring homozygous or heterozygous DUSP12 gene variant. This DUSP12 variant rs781449881 causes a codon change from Pro81 residue to Arg [47]; this residue is in the phosphatase domain of DUSP12. This variant may inhibit DUSP12 phosphatase activity by altering a non-charged proline into a positive-charged arginine, promoting human autoimmunity. These findings suggest that DUSP12 may play an important role in the prevention of autoimmune diseases.

### DUSP14 (MKP6)

**Fig. 6:**
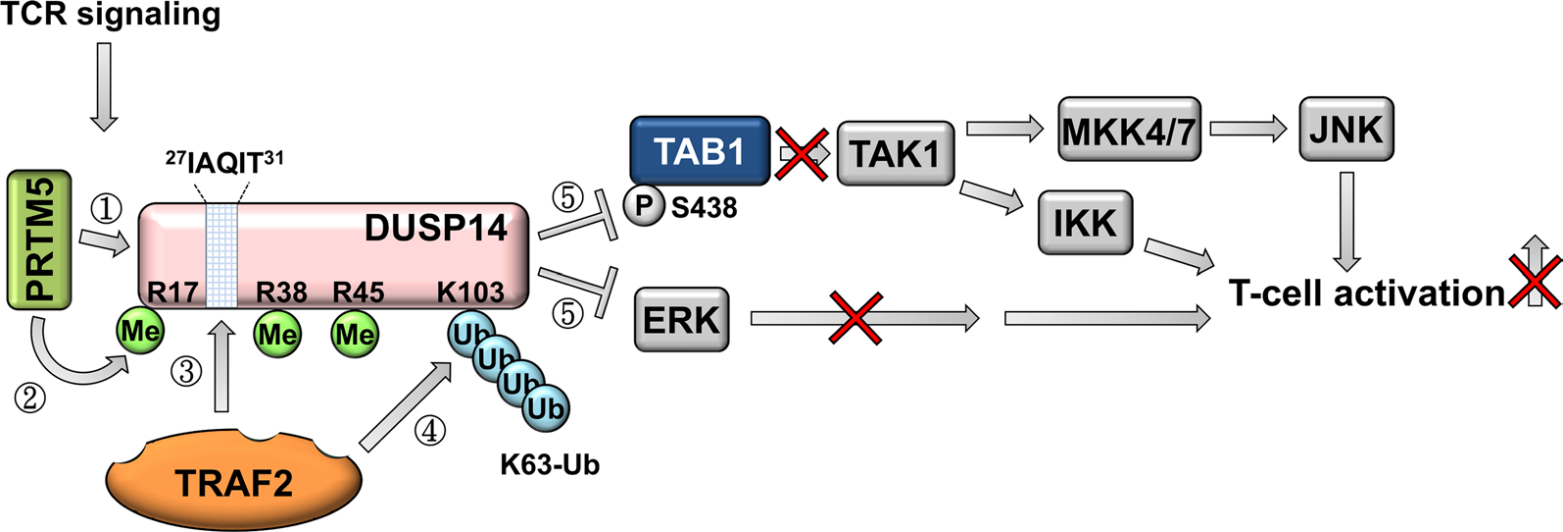
DUSP14 inhibits T-cell activation by dephosphorylating TAB1. During TCR signaling stimulation, the methyltransferase PRMT5 binds to DUSP14 (step 1) and methylates DUSP14 at Arg17, Arg38, and Arg45 residues (step 2). The TRAF2-binding motif (^27^IAQIT^31^) of the methylated DUSP14 interacts with the E3 ubiquitin ligase TRAF2 (step 3), which induces K63-linked ubiquitination of DUSP14 at Lys103 residue (step 4). DUSP14 phosphatase activity is induced after TRAF2-mediated K63-linked ubiquitination. Activated DUSP14 inhibits ERK activation (step 5) and dephosphorylates TAB1 at Ser438 residue (step 5), leading to the suppression of T-cell activation. Adapted from [99]

DUSP14 (also named MAPK phosphatase 6, MKP6) is a negative regulator of TCR signaling that dephosphorylates and inactivates ERK and JNK [26]. DUSP14 phosphatase activity is induced by PRMT5-mediated methylation at Arg17, Arg38, and Arg45 residues [99] and subsequent TRAF2-mediated K63-linked ubiquitination at Lys103 residue [100] (Fig. 6). Conversely, overexpression of the dominant-negative DUSP14 mutant in human primary T cells induces the activation of ERK and JNK [26]. Consistently, DUSP14-deficient mice show an enhancement of T-cell activation and proliferation [29]. Besides targeting ERK, DUSP14 directly inactivates TAB1 by dephosphorylating its Ser438 residue, inhibiting the TCR-stimulated activation of TAK1 and downstream JNK/IKK in human Jurkat T cells upon TCR signaling (Fig. 6). Moreover, DUSP14-deficient mice show increased serum levels of IL-17A and IFN-γ, as well as more severe paralysis compared to those of wild-type mice during the induction of EAE [29]. Transcriptomic meta-analysis data of human whole blood samples show that DUSP14 gene expression is downregulated in axial spondyloarthritis/AS patients [101]. In contrast, DUSP14 mRNA levels are induced and correlated with TNF-α levels in the peripheral T cells of AS patients [77]. These results suggest that DUSP14 is induced in T-cell-mediated autoimmune responses; however, the role of DUSP14 in human AS pathogenesis needs to be clarified.

### DUSP16 (MKP7)

DUSP16 (also named MAPK phosphatase 7, MKP7) is a typical DUSP. DUSP16 specifically dephosphorylates and inactivates the MAPK JNK in African green monkey COS-7 cells and murine T cells [102–104]. In contrast, DUSP16 is phosphorylated by the MAPK ERK at Ser446 residue, preventing DUSP16 ubiquitination and proteasomal degradation [102, 105]. The stabilized DUSP16 then binds to JNK-interacting protein-1 (JIP-1), leading to JNK inactivation [103]. Besides JNK, DUSP16 modestly reduces tyrosine phosphorylation of ERK in murine CD4^+^ T cells [106]. DUSP16 deficiency results in a reduction of Th17 differentiation but an increase of IL-2 production in murine CD4^+^ T cells [106]. Notably, the decreased Th17 differentiation caused by DUSP16 deficiency cannot be reversed by inhibiting JNK. DUSP16 deficient (gene-trapped) mice are resistant to Th17-mediated EAE induction, suggesting that DUSP16 plays a positive role in Th17-mediated autoimmune responses. Paradoxically, DUSP16 knockdown in murine dendritic cells results in an elevation of antigen-presenting ability, leading to an increased proliferation of Th17 cells isolated from mice with the induction of experimental autoimmune uveitis [107]. DUSP16 3’-UTR is targeted by miR-338-3p in murine dendritic cells, resulting in downregulation of DUSP16 mRNA levels [107]. The data suggest that DUSP16 inhibits dendritic cell-mediated Th17 immune responses. Collectively, the intrinsic roles of DUSP16 in Th1, Th17, and dendritic cell differentiation are complex and need to be further investigated using conditional knockout (cKO) mice. The role of DUSP16 in human autoimmune diseases remains to be validated.

### DUSP22 (JKAP, JSP1)

**Fig. 7:**
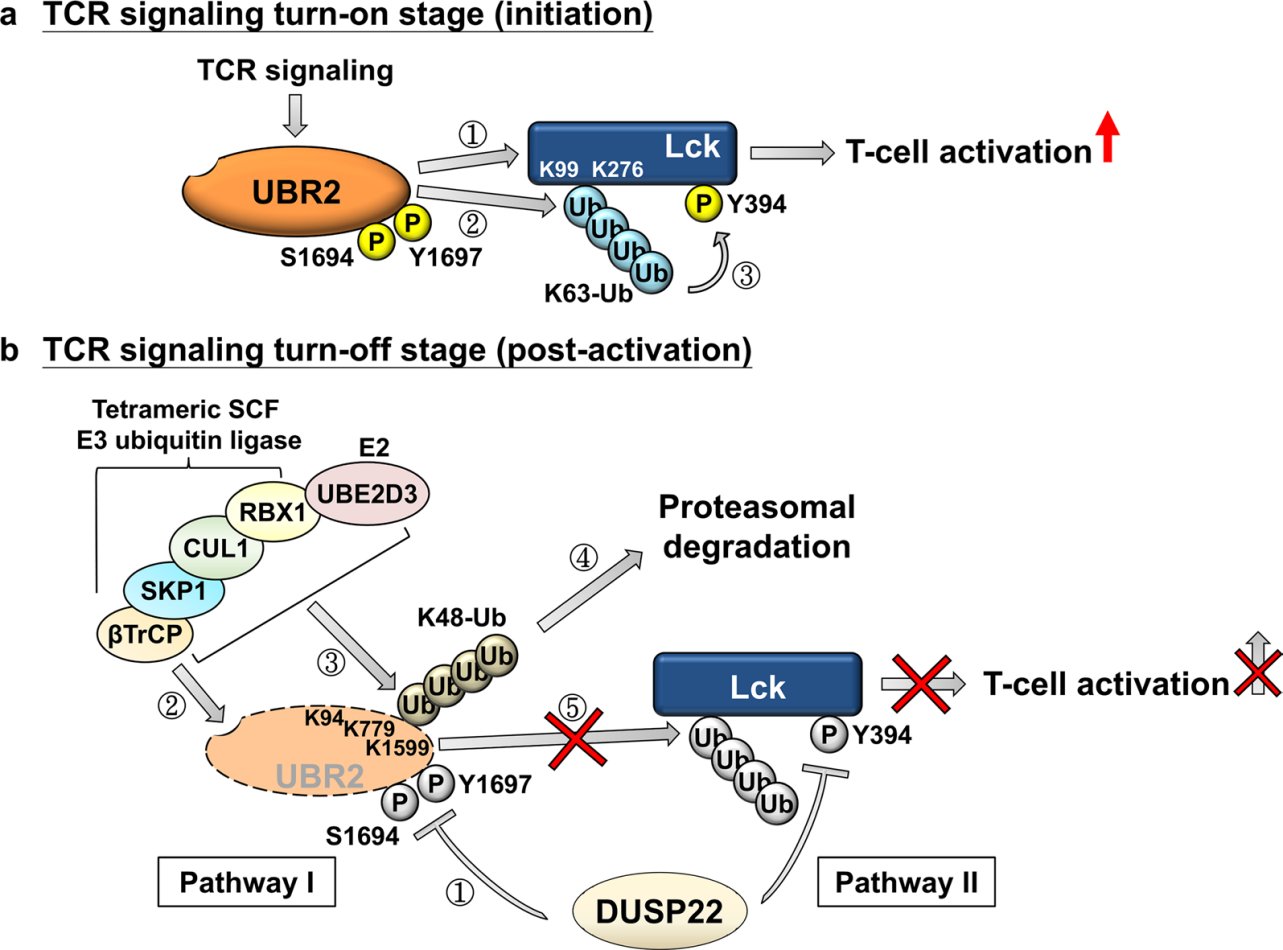
DUSP22 inhibits T-cell activation by dephosphorylating UBR2 and Lck. **a** The E3 ubiquitin ligase UBR2 is phosphorylated at Ser1694 and Tyr1697 residues in TCR signaling turn-on (early activation) stage. Phosphorylated UBR2 interacts with the tyrosine kinase Lck (step 1) and mediates Lck K63-linked ubiquitination at Lys99 and Lys276 residues (step 2), leading to Lck Tyr394 autophosphorylation (step 3) and subsequent T-cell activation. **b** DUSP22 abolishes Lck activation in TCR signaling turn-off (post activation) stage by two pathways. In the first pathway, DUSP22 induces UBR2 proteasomal degradation. DUSP22 dephosphorylates UBR2 at Ser1694 and Tyr1697 residues (step 1), resulting in the interaction ofUBR2 with SCF E3 ubiquitin ligase complex (step2). SCF E3 ubiquitin ligase complex induces UBR2 K48-linked ubiquitination (step 3) at Lys94, Lys779, and Lys1599 residues, leading to UBR2 proteasomal degradation (step 4). As a result, UBR2-induced Lck autoactivation is blocked (step 5). In the second pathway, DUSP22 inactivates Lck by directly dephosphorylating Lck at Tyr394, resulting in the inhibition of Lck activation and subsequent T cell activation. Adapted from [30]

DUSP22 (also named JNK pathway-associated phosphatase, JKAP or JNK stimulatory phosphatase-1, JSP1 [108, 109]) is a negative regulator of TCR signaling by inhibiting Lck activity through two different pathways (Fig. 7). In TCR signaling turn-on (initiation) stage (Fig. 7a), the E3 ubiquitin ligase UBR2 is phosphorylated at Ser1694 and Tyr1697 residues by an unknown serine/threonine kinase. The phosphorylated UBR2 interacts with Lck and induces Lck K63-linked ubiquitination at Lys99/Lys276 residues, leading to Lck Tyr394 autophosphorylation and subsequent T-cell activation [30]. In TCR signaling turn-off (post-activation) stage, DUSP22 suppresses TCR-induced Lck activation through 2 different pathways (Fig. 7b). In the pathway I, DUSP22 indirectly inhibits Lck activation by downregulating UBR2, an Lck activator. Mechanistically, DUSP22 dephosphorylates UBR2 at Ser1694 and Tyr1697 residues, leading to UBR2 proteasomal degradation and Lck inactivation [30]. In the pathway II, DUSP22 directly dephosphorylates Lck at Tyr394 residue, resulting in abolishment of Lck activation and subsequent T-cell-mediated inflammation [31]. Conversely, DUSP22 KO mice show severe symptoms after EAE induction compared to wild-type mice. Moreover, aged DUSP22 KO mice spontaneously develop inflammation in liver, kidney, and lung, as well as induction of serum autoantibodies [31, 77]. DUSP22 KO mice also manifest AS-like symptoms [77]. Consistently, T-cell-specific transgenic mice expressing the dominant-negative DUSP22-C88S mutant also show T-cell hyperactivation, inflammation, and autoimmune nephritis [110]. As expected, Lck Tyr394 phosphorylation and K63-linked ubiquitination are induced in T cells of DUSP22 KO mice but are abolished in DUSP22/UBR2 dKO mice [30]. Multi-organ inflammation of DUSP22 KO mice is also greatly alleviated in DUSP22/UBR2 dKO mice. These findings indicate that DUSP22 downregulation and UBR2 induction promote T-cell-mediated inflammation and autoimmune disease in mice.

DUSP22 protein levels are greatly decreased in the peripheral blood T cells of SLE patients compared to those of healthy individuals [30, 110]. The percentage of Tyr394-phosphorylated Lck containing T cell is elevated in SLE patients [110]. UBR2 protein levels and K63-linked-ubiquitinated Lck levels are also increased in T cells of SLE patients [30]. DUSP22 protein levels in T cells are inversely correlated with daily urinary protein levels in SLE patients, suggesting that DUSP22 downregulation in T cell is a non-invasive biomarker for the severity of LN [110]. The diagnostic power of DUSP22 downregulation is higher than serum C3, C4 complements, and anti-dsDNA antibody levels. Moreover, SLE patients with lower DUSP22 expression levels in peripheral blood T cells show poor renal outcome, indicating that downregulation of DUSP22 is a prognostic biomarker of SLE nephritis [110]. Collectively, the data indicate that downregulation of DUSP22 in T cells leads to T cell-mediated inflammation and SLE nephritis.

Besides SLE, DUSP22 is involved in another autoimmune disease, RA. Serum DUSP22 levels are downregulated in RA patients compared to healthy individuals [111]. RA patients who are responsive to triple conventional disease-modifying anti-rheumatic drugs therapy show an increase of serum DUSP22 levels [112]. DUSP22 mRNA levels are also decreased in the synovium of RA patients compared to those of trauma patients [111]. These findings suggest that DUSP22 levels are inversely correlated with RA severity. Genome-wide DNA methylation analyses of peripheral blood mononuclear cells (PBMCs) from RA patients show that 10 sites at the DUSP22 promoter region are hypomethylated and associated with erosive RA [113]. Another study shows that the hypomethylation in only one CpG site of the DUSP22 promoter from the plasma DNA samples is associated with human RA severity; however, its significance is unclear. In contrast, one report using genome-wide DNA methylation sequencing profile and then bisulfate pyrosequencing validation identifies 4 RA-associated hypermethylation sites at the DUSP22 promoter region from T cells of RA patients [114], suggesting that downregulation of DUSP22 gene expression occurs in T cells of RA patients. Thus, downregulation of DUSP22 in T cell may be a potential biomarker of RA; however, functional changes of these hypomethylation sites have not been validated in these two publications.

DUSP22 mRNA levels are decreased in peripheral T cells of AS patients compared to those of healthy individuals [77]. The mRNA levels of the inflammatory cytokine TNF-α are inversely correlated with DUSP22 mRNA levels in T cells of AS patients. The percentages of IL-17A- and IFN-γ-secreting T cells are also increased in the peripheral blood of AS patients. Moreover, DUSP22 mRNA levels in T cells of AS patients are inversely correlated with disease activity based on CRP levels, erythrocyte sedimentation rate, and the Bath Ankylosing Spondylitis Disease Activity Index (BASDAI) [77]. These results suggest that DUSP22 downregulation in T cell contributes to the pathogenesis of human AS.

Genome-wide DNA methylation profile shows that 8 hypermethylated sites at DUSP22 promoter region in naïve CD4^+^ T cells of patients with SS compared to those of healthy individuals [115], suggesting that DUSP22 transcription may be downregulated in T cells of SS patients.

Taken together, downregulation of the TCR negative regulator DUSP22 leads to autoimmune diseases, such as SLE, RA, AS, and SS by inducing T cell activation. Thus, inducing DUSP22 levels may be a new therapeutic strategy for autoimmune diseases. It would be valuable to study the regulatory mechanism of DUSP22 downregulation in autoimmune diseases.

### DUSP23 (VHZ)

DUSP23 (also named VH1-related protein Z member, VHZ) is an atypical DUSP that contributes to placental development and neuronal differentiation [116, 117]. Real-time PCR analyses using individual human tissues show that DUSP23 mRNA is expressed in multiple organs/tissues of fetus but only in the colon and testis of adults [27]. DUSP23 dephosphorylates ERK1 but does not dephosphorylate JNK or p38 in vitro [27]. DUSP23 overexpression in COS-7 cells induces the activation of MKK4 and MKK6, as well as the downstream molecules JNK and p38 in a phosphatase activity-independent manner [118]. Genome-wide association studies show that several lupus predisposing gene loci are located on human chromosome 1 (1q21-23), which contains the DUSP23 gene locus [119]. Moreover, DUSP23 mRNA levels are increased in peripheral blood CD4^+^ T cells of SLE patients compared to those of healthy individuals. DUSP23 mRNA levels are higher in SLE patients with severe symptoms compared to those of patients with mild symptoms [119]. Thus, upregulation of DUSP23 in T cells may contribute to SLE pathogenesis.

## The roles of DUSPs in cell signaling of allergic diseases

DUSP1, DUSP2, DUSP8, and DUSP14 are involved in pathogenesis of allergic diseases, such as allergic asthma and atopic dermatitis (Table 4). Allergic diseases are triggered by allergens, inducing immune hyperreactivity and inflammation [120]. Increasing prevalence of allergic diseases is one of the major global health issues [121]. To study and understand the regulatory mechanism of allergic diseases would be helpful for alleviating the health and social economic burdens induced by this rising issue.

### DUSP1 (MKP1, PTPN10)

DUSP1 mRNA levels are downregulated in the primary nasal epithelial cells isolated from allergy patients upon in vitro stimulation of house dust mite [122]. Similarly, DUSP1 mRNA levels are also decreased in bronchial epithelial cells of allergic rhinitis and asthma patients [123]. In addition, DUSP1 downregulation in the lung tissue is associated with steroid resistance in ovalbumin-induced asthma mice [124]. Knockdown of DUSP1 induces mRNA levels of the proinflammatory cytokines IL-6 and IL-8 in human NCI-H292 airway epithelial cells upon house dust mite or poly(I:C) stimulation [125], suggesting that DUSP1 downregulation in epithelial cells may induce inflammatory/allergic responses.

### DUSP2 (PAC1)

DUSP2 negatively regulates Th2-mediated allergic inflammation [50]. Female mice or T-cell-specific androgen receptor cKO mice show more pronounced allergic asthma and increased Th2 cytokine levels during the induction of ovalbumin or house dust mite-induced asthma [50, 126]. DUSP2 mRNA levels are decreased in differentiated Th2 cells of androgen receptor cKO mice [50]. Conversely, DUSP2 overexpression suppresses IL-4 production of differentiated Th2 cells. Mechanistically, androgen binds to an atypical androgen-response element within the DUSP2 promoter, promoting DUSP2 transcription and subsequently inhibiting Th2 differentiation [50]. Collectively, that DUSP2 plays a negative role in Th2-mediated allergic asthma.

### DUSP8 (M3/6)

**Fig. 8:**
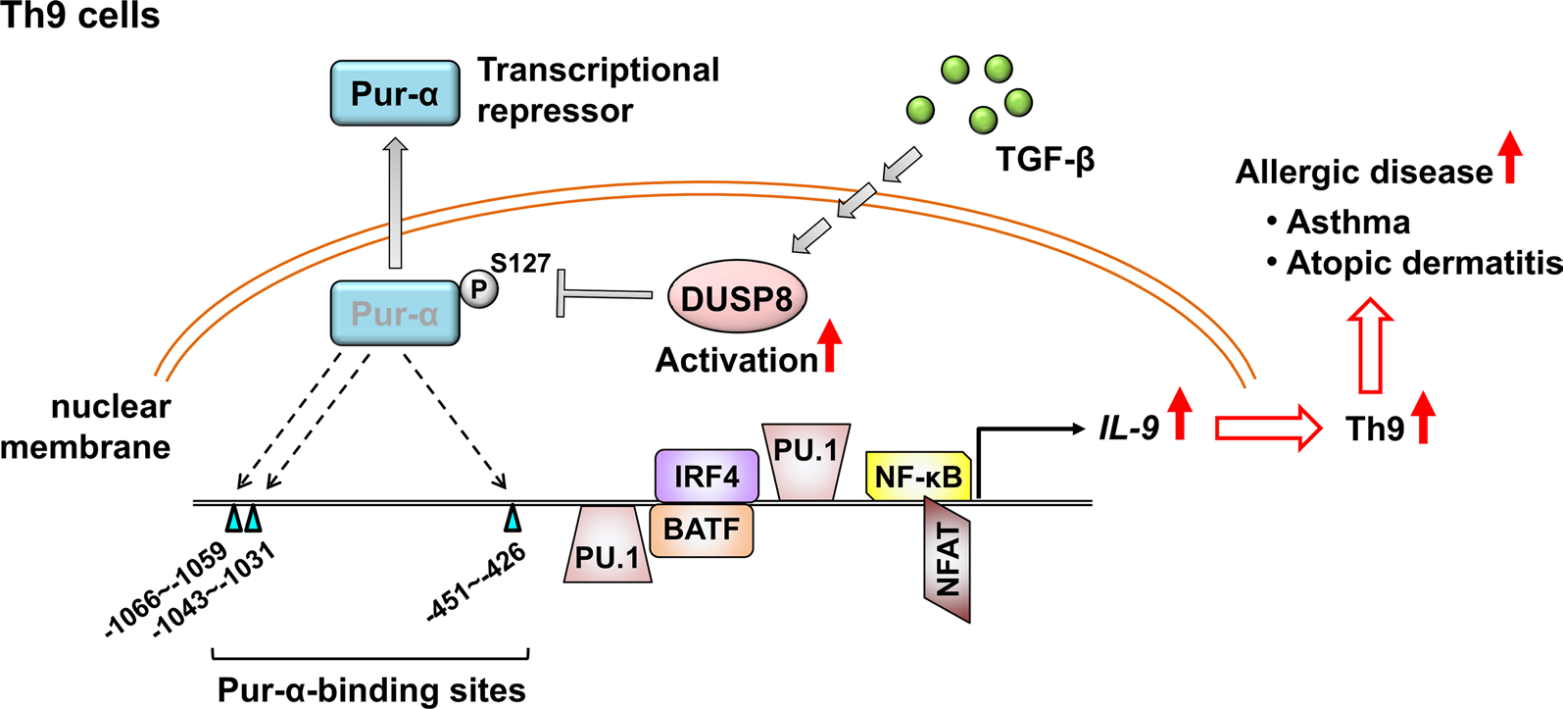
DUSP8 induces Th9 differentiation and Th9-mediated allergic diseases by dephosphorylating Pur-α. The transcriptional repressor Pur-α binds to three sites of the IL-9 promoter in T cells, leading to the suppression of IL-9 transcription. TGF-β signaling induces DUSP8 phosphatase activity in T cells. DUSP8 directly dephosphorylates Pur-α at Ser127 residue, resulting in nuclear export of Pur-α. Loss of Pur-α binding on the IL-9 promoter results in the induction of IL-9 transcription and Th9 differentiation, contributing to Th9-mediated allergic diseases. Adapted from [32]

DUSP8 (also named Homologue of vaccinia virus H1 phosphatase gene clone 5, hVH5, or M3/6 [127]) is highly expressed in T cells and platelet [128]. DUSP8 specifically dephosphorylates and inactivates JNK, but not ERK or p38 [127, 129]. Th9 (CD3^+^CD4^+^IL-9^+^) cells enhance allergic inflammation and airway hyperreactivity [130]. Phosphatase activity of DUSP8 is induced by Th9 differentiation cytokine TGF-β; DUSP8 cKO suppresses Th9 in vitro differentiation [32]. Overexpression of DUSP8, but not phosphatase-dead mutant, enhances IL-9 promoter activity in human Jurkat T cells, suggesting that DUSP8 phosphatase activity is required for IL-9 gene transcription. Analyses using mass spectrometry analysis, co-immunoprecipitation analysis, proximity ligation assay, and fluorescence resonance energy transfer show that DUSP8 directly interacts with the DNA-binding protein Pur-α, which binds to the promoter of IL-9 and suppresses IL-9 transcription. This suppression is reversed by overexpression of DUSP8, indicating that DUSP8 inhibits Pur-α-induced transcriptional repression of IL-9. Mechanistically, DUSP8 dephosphorylates Pur-α at Ser127 residue, leading to the translocation of Pur-α from nucleus to cytoplasm [32]. T cell-specific DUSP8 cKO mice are resistant to ovalbumin-induced asthma, whereas the symptoms are restored by Pur-α heterozygous cKO. Remarkably, DUSP8 protein is highly expressed in the peripheral blood T cells of asthma patients compared to other DUSPs by mass proteomics. DUSP8 co-exists with IL-9 in peripheral T cells of asthma patients. Moreover, the frequency of DUSP8-positive T cells in the peripheral blood leukocytes of asthma patients is much higher than those in healthy individuals. The DUSP8-Pur-α protein complex is colocalized and induced in the cytoplasm T cells from asthma and atopic dermatitis patients. Taken together, DUSP8 induces the differentiation of Th9 cells by dephosphorylating the transcriptional repressor Pur-α and inducing IL-9 transcription, leading to the pathogenesis of allergic asthma and atopic dermatitis [32] (Fig. 8).

### DUSP14 (MKP6)

DUSP14 mRNA and protein levels are decreased in the lung tissue of asthma mice and IL-13-stimulated human BEAS-2B bronchial epithelial cells [131]. DUSP14 overexpression in mice by viral transduction results in the decrease of IL-4 levels, GATA3 levels, Th2 cells, eosinophils, and neutrophils in the lung tissue of ovalbumin-induced asthma mice. Moreover, DUSP14 overexpression decreases mRNA and protein levels of mucin MUC5AC in the lung tissue of asthma mice. Similarly, levels of IL-4, IL-5, and MUC5AC are deceased by DUSP14 overexpression in IL-13-stimulated BEAS-2B bronchial epithelial cells [131]. These findings suggest that DUSP14 downregulation is involved in the pathogenesis of allergic asthma.

## The roles of DUSPs in cell signaling of IBDs

IBD is an idiopathic disorder that shows chronic inflammation in the intestinal tract. Ulcerative colitis (UC) and Crohn’s disease (CD) are the two most common IBDs. UC affects the large intestine, whereas CD affects any part of the intestinal tract. DUSP2, DUSP6, DUSP11, DUSP16, DUSP22, and DUSP28 are associated with IBD by regulating immune cell function, epithelial cell integrity, and gut microbe maintenance (Table 5).

### DUSP2 (PAC1)

**Fig. 9:**
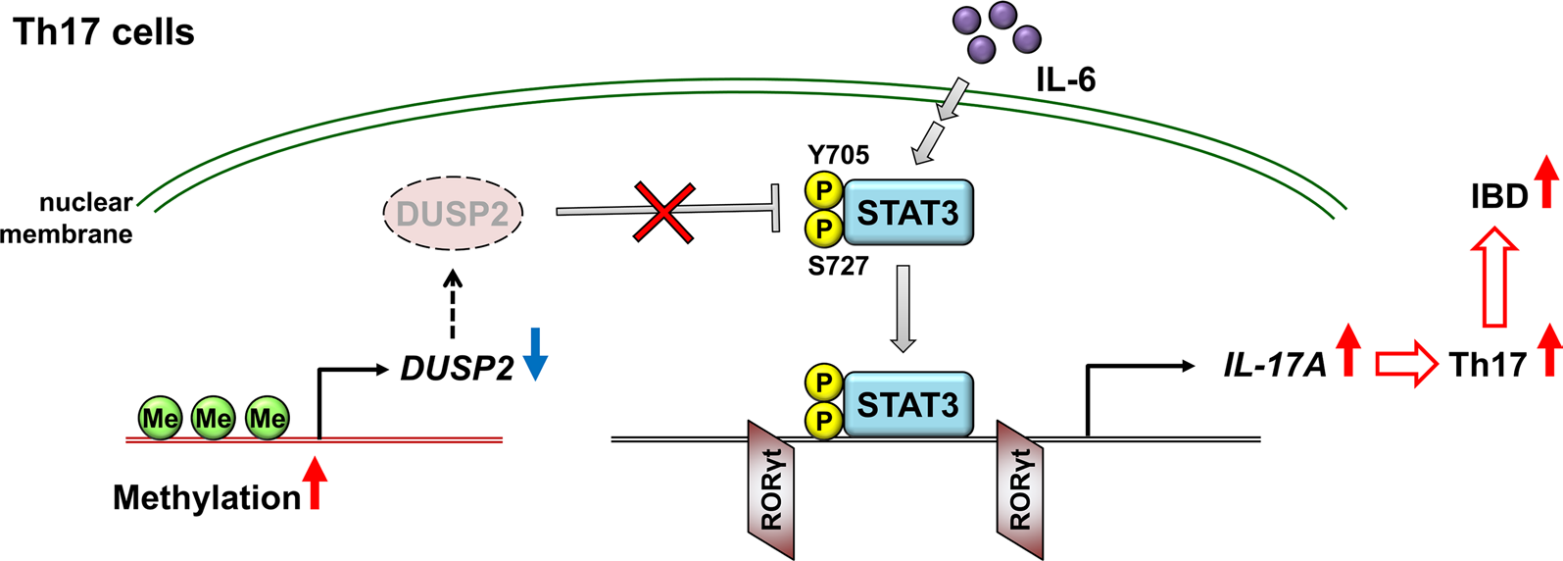
DUSP2 deficiency contributes to Th17-mediated IBD by dephosphorylating STAT3. The increased methylation frequency of DUSP2 promoter region results in the decrease of DUSP2 protein levels in UC patients. DUSP2 downregulation results in the enhancement of IL-6-mediated STAT3 Tyr705/Ser727 phosphorylation and activation. Activated STAT3 binds to the promoter region of IL-17A and induces IL-17A transcription, contributing to Th17 differentiation and IBD development

DUSP2 KO results in an induction of murine Th17 differentiation [33]. DUSP2 KO mice display an exacerbated colitis symptom with Th17 induction in lymph nodes upon dextran sulfate sodium (DSS) administration [33]. T cells of IBD patients show an induction of activated STAT3 [132, 133], which is essential for IL-17A transcription and Th17 differentiation. Consistently, phosphorylated STAT3 levels are greatly induced in the colon tissue of DUSP2 KO mice upon DSS injection [33]. Mechanistically, DUSP2 directly dephosphorylates STAT3 at Tyr705 and Ser727 residues, leading to inactivation of STAT3. Overexpression of DUSP2 reduces STAT3-mediated IL-17A transcription. Furthermore, methylation frequencies of the DUSP2 promoter region are increased in UC patients compared to those of healthy individuals. As the result, DUSP2 mRNA levels are decreased in PBMCs of UC patients [33] (Fig. 9). Collectively, DUSP2 downregulation induces STAT3-mediated Th17 differentiation, leading to the development of IBD.

### DUSP6 (MKP3)

T cell proliferation and Th1 differentiation are induced, whereas Th17 differentiation and Treg function are reduced by DUSP6 KO [134]. DUSP6 KO mice show exacerbated colitis in the enterocolitis murine model induced by IL-10 KO. The IFN-γ production and ERK activation in T cells of the symptomatic mice are increased by DUSP6 KO. Notably, the accelerated colitis by DUSP6 KO is attenuated by the treatment of the MEK (ERK activator) inhibitor PD0325901 [134]. These findings suggest that DUSP6 may suppress Th1-mediated colitis by inhibiting ERK activation.

Transcriptomics and single-cell RNA sequencing (scRNA-seq) analyses show that DUSP6 mRNA levels are increased in the inflamed colonic mucosa and epithelial cells, but not immune or stromal cells of UC patients [135]. DUSP6 deficiency results in a decrease of inflammatory immune cells in the murine intestine [136]. Moreover, DUSP6 KO mice display ameliorated DSS-induced colitis and robust intestinal epithelium integrity compared to wild-type mice [135, 137]. Interestingly, the epithelial-to-mesenchymal transition (EMT) pathway and glucose metabolism are reduced in DUSP6 KO human Caco-2 epithelial cells, whereas oxidation and lipid metabolism are induced [135]. These results suggest that DUSP6 deficiency contributes to metabolic switch of gut epithelial cells, leading to the adjustment of the micro-environment for gut microbiota. Furthermore, a Gram-negative obligate anaerobe (*Duncaniella muris*, named NHRI-C1-K-H-1-87) is identified as an enriched bacterium in the feces of DUSP6 KO mice. Notably, treatment of NHRI-C1-K-H-1-87 alleviates the DSS-induced colitis in germ-free wild-type recipient mice [135]. Collectively, downregulation of DUSP6 prevents colitis by strengthening the intestinal epithelium and maintaining the probiotics.

### DUSP11 (PIR1)

Transcriptomic analysis shows that DUSP11 mRNA levels are downregulated in the sigmoid colon or the terminal ileum of both UC and CD patients compared to those of healthy individuals [138]. Notably, DUSP11 downregulation does not occur in patients with other colonic diseases, such as infectious diarrhea, irritable bowel syndrome, or other forms of gastrointestinal inflammation [138]. These data implicate that DUSP11 may be involved in IBD.

### DUSP16 (MKP7)

Transcriptome expression patterns of individual 145 human IBD-associated genes in human HT-29 colonic epithelial cells are analyzed and compared to those of the IBD causative gene KSR1 (kinase suppressor of Ras 1) [139]. KIR1 induces EMT-like phenotypes in human colorectal cancer cell lines [140]. The similarity analysis shows that the genes upregulated by DUSP16 is correlated with that by KSR1 in HT-29 epithelial cells [139]. In addition, TNF-α-induced JNK activation in HT-29 cells is abolished by DUSP16 overexpression, while TNF-α-induced ERK or p38 activation is modestly reduced. These results implicate that DUSP16 may be associated with the attenuation of TNF-α signaling in IBD.

### DUSP22 (JKAP, JSP1)

DUSP22 mRNA and protein levels are decreased in the intestine mucosa of active IBD (both UC and CD) patients, and DUSP22 protein levels are inversely correlated with the disease activity [141]. The mRNA levels of DUSP22 are also negatively correlated with mRNA levels of the inflammatory cytokines TNF-α, INF-γ, IL-17, and IL-10 in the intestine mucosa of IBD patients. T-cell activation, proliferation, and Th1/Th17 differentiation of human primary T cells are reduced by DUSP22 overexpression but induced by DUSP22 siRNA knockdown [141]. Collectively, DUSP22 downregulation in T cells may contribute to the progression of human IBD.

### DUSP28 (VHP)

DUSP28 (also named VH1-dual specificity phosphatase family protein, VHP) is an atypical DUSP. Genome-wide association study (GWAS) shows that DUSP28 is associated with UC [142]. Cross-tissue and single-tissue transcriptome-wide association studies show that DUSP28 transcripts are negatively correlated with UC [142]. It would be interesting to study DUSP28 downregulation-mediated pathological mechanism of human UC.

## The roles of DUSPs in obesity, diabetes, and other metabolic diseases

Based on the statistics in 2022 from World Health Organization, 43% of adults are overweight and 16% of adults are obese [143]. Obesity is a risk factor for inflammatory and metabolic diseases, such as diabetes mellitus, which is clinically characterized by hyperglycemia [144]. Diabetes mellitus hyperglycemia leads to multiple complications, including cardiovascular disease, diabetic nephropathy, diabetic neuropathy, and diabetic retinopathy. Type 1 diabetes is an autoimmune disease with deficiency of insulin-producing β-cells in the pancreas [144], while type 2 diabetes (T2D) is characterized by insulin resistance in the insulin-responsive tissues (adipocytes, hepatocytes, and muscle cells) [145]. Obesity and T2D also contribute to non-alcoholic fatty liver disease (NAFLD), which is characterized by accumulation of hepatic lipid droplets (non-alcoholic fatty liver) and subsequent non-alcoholic steatohepatitis (NASH). Multiple DUSPs (including DUSP1, DUSP3, DUSP4, DUSP5, DUSP6, DUSP7, DUSP8, DUSP9, DUSP10, DUSP12, DUSP14, DUSP15, DUSP16, DUSP22, and DUSP26) play important roles in the pathogenesis of obesity, diabetes, or other metabolic diseases (Table 6).

### DUSP1 (MKP1, PTPN10)

DUSP1 mRNA levels are decreased in the kidney of diabetic nephropathy patients [146]. Similarly, DUSP1 mRNA and protein levels are decreased in the kidney of diabetic nephropathy rats [146]. Conversely, DUSP1 overexpression ameliorates nephropathy in diabetic mice [147]. These results suggest that DUSP1 downregulation is associated with the pathogenesis of diabetic nephropathy.

DUSP1 protein levels are increased in the PBMCs and subcutaneous adipose tissue of human obese participants, as well as in the liver tissue of high-fat diet (HFD)-fed mice [148, 149]. As expected, p38 activation is decreased in the PBMCs and the subcutaneous adipose tissue of obese patients [148]; conversely, activation of p38 and JNK is increased in the insulin-responsive tissues of DUSP1 KO mice [150]. Normal chow diet-fed DUSP1 KO mice show less body weight, fat mass, hepatic triglycerides, and hepatic lipid accumulation [150]. Moreover, PPAR-α activity and fatty acid oxidation are increased in murine primary hepatocytes derived from DUSP1 KO mice. Furthermore, DUSP1 KO mice are resistant to HFD-induced obesity; however, HFD-fed DUSP1 KO mice display glucose intolerance. The unexpected glucose intolerance in DUSP1 KO mice may be due to the hyperactivation of JNK and p38, which inhibit IRS1 activation during insulin signaling [150]. Notably, the glucose uptake of murine 3T3-L1 adipocytes is modestly decreased by DUSP1 overexpression [151]. The complex roles of DUSP1 in glucose homeostasis should be investigated using individual tissue-specific DUSP1 cKO.

Interestingly, hepatocyte-specific DUSP1 cKO HFD-fed mice show an increase of body weight and glucose intolerance, but a decreased of hepatic steatosis [149]. The liver tissue of DUSP1 cKO mice displays decreased levels of FGF21, which is responsible for energy expenditure and weight loss [149]. The increase of body weight could be due to the reduction of hepatic FGF21 in DUSP1 KO mice; however, the reason for the reduction of hepatic steatosis in DUSP1 KO mice remains unclear.

DUSP1 mRNA levels are increased in the liver tissue of patients with obese steatosis and obese NASH [152]. Consistently, DUSP1 protein levels are increased in the primary hepatocytes of mice fed with choline-deficient L-amino acid-defined (CDAA) diet [152] or HFD [149]. Interestingly, CDAA-induced hepatic lipid accumulation, fibrosis, and steatosis are alleviated in hepatocyte-specific DUSP1 cKO mice [152]. The main features of NASH activation, apoptosis and autophagy of hepatocytes, are also decreased in DUSP1 cKO mice. Mechanistically, p38 inactivation by DUSP1 reduces AMPK activation, leading to caspase 3-induced apoptosis and caspase 6-induced autophagy of hepatocytes [36, 54, 55]. In addition, DUSP1 overexpression induces the nuclear translocation of the AMPK activator-LKB1 (also known as STK11) in human HepG2 hepatocytes [152]. It would be interesting to study whether DUSP1 inhibits AMPK activation by inducing LKB1 nuclear translocation. These findings suggest that hepatic DUSP1 is a biomarker and therapeutic target of NASH.

### DUSP3 (VHR)

DUSP3 mRNA levels in the liver tissue are decreased in 18-month-old HFD-fed mice compared to those of chow diet-fed mice [153]. HFD-fed DUSP3 KO mice display the induction of body weight, liver mass, serum cholesterol levels, hepatic lipid accumulation, insulin resistance, and liver injury compared to those of HFD-fed wild-type mice. Moreover, the severity of diethylnitrosamine-induced NAFLD and hepatocellular carcinoma are enhanced by DUSP3 KO [153]. These results suggest that downregulation of DUSP3 contributes to obesity and NAFLD.

### DUSP4 (MKP2)

DUSP4 protein levels are increased in the liver tissue of obese NASH patients and in the insulin-responsive tissues of HFD-induced mice [73]. HFD-fed DUSP4 KO mice display the reduction of body weight, fat mass, food intake, insulin resistance, and hepatic steatosis compared to those of HFD-fed wild-type mice. Interestingly, the protein kinase B (AKT) phosphatase-PTEN [154, 155] is decreased in the liver tissue of HFD-fed DUSP4 KO mice, resulting in the activation of AKT. Additionally, DUSP4 KO mice display increased serum levels of insulin-like growth factor 1 (IGF-1), which subsequently induces the IGF-1−AKT pathway in the liver of DUSP4 KO mice [73]. These findings suggest that downregulation of DUSP4 results in the suppression of diet-induced obesity and NAFLD.

### DUSP5 (VH3)

DUSP5 mRNA and protein levels are decreased in the heart tissue of the type 1 diabetes murine model OVE26 transgenic mice [156], which also develop diabetes-induced cardiac dysfunction [157]. Histone deacetylase 3 (HDAC3) upregulation is involved in the pathogenesis of heart failure in mice [158–160]. The treatment of a HDAC3 inhibitor, RGFP966, ameliorates diabetes-induced cardiac hypertrophy, cardiac fibrosis, cardiac inflammation, and insulin resistance in OVE26 transgenic mice; while DUSP5 levels and ERK activation are decreased in the heart tissue of RGFP966 treated mice [156]. Chromatin immunoprecipitation results show that inhibition of HDAC3 enhances the binding of acetylated histone H3 to the DUSP5 promoter [156]. Taken together, these results suggest that HDAC3-mediated DUSP5 downregulation may promote the progression of diabetic cardiomyopathy.

### DUSP6 (MKP3)

**Fig. 10:**
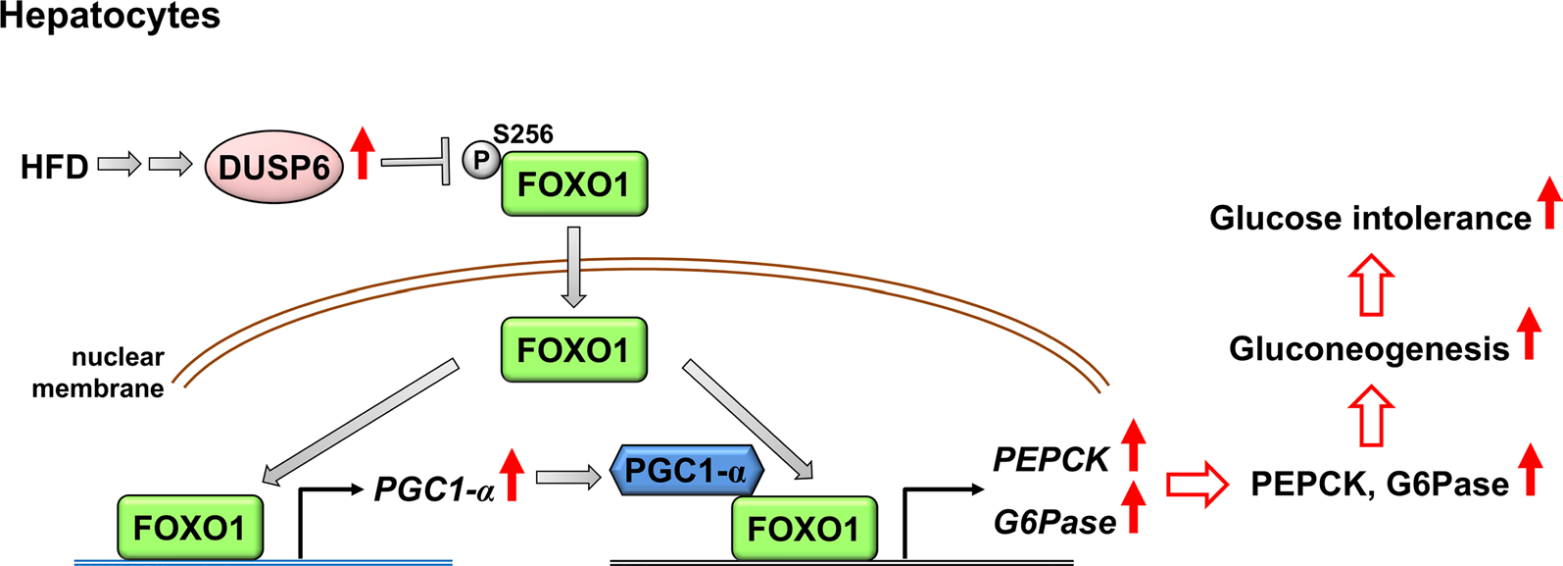
DUSP6 mediates HFD-induced glucose intolerance by dephosphorylating FOXO1. DUSP6 protein levels are increased in the liver of HFD-fed obese mice. DUSP6 dephosphorylates FOXO1 at Ser256 residue, promoting the nuclear translocation of FOXO1 and the subsequent enhancement of PGC1-α transcription. FOXO1 further cooperates with PGC1-α to induce the transcription of two gluconeogenesis-related genes, *PEPCK* and *G6Pase*, leading to gluconeogenesis and glucose intolerance

DUSP6 protein levels are increased in the liver of HFD-induced obese mice [34, 161]. Adenovirus-transduced DUSP6 inhibits ERK activation in the liver tissue and increases blood glucose/insulin levels in lean mice [34]. Conversely, DUSP6 knockdown results in the reduction of palmitic acid and oleic acid-stimulated lipid accumulation in human HepG2 or Huh7 hepatocytes [162]. In addition, blood glucose levels of HFD-induced or ob/ob (*Lep*^*ob*^) mice are significantly decreased by DUSP6 shRNA knockdown or DUSP6 KO [34, 163]. The insulin resistance-related islet hyperplasia in HFD-fed mice is inhibited by DUSP6 KO [163]. Notably, the body weights of these mice are reduced by DUSP6 homozygous KO [163] but not affected by DUSP6 shRNA knockdown [34] or DUSP6 heterozygous KO [163]. Interestingly, DUSP6 induces gluconeogenesis in the liver tissue of mice and in rat Fao hepatoma cells [34, 161]. Mechanistically, DUSP6 dephosphorylates the transcription factor FOXO1 at Ser256 residue, promoting the nuclear translocation of FOXO1 and subsequent transcription of the transcription coactivator PPAR-γ coactivator 1 α (PGC-1α). FOXO1 then cooperates with PGC-1α to induce transcription of gluconeogenesis-related genes-PEPCK and glucose 6-phosphatase (G6Pase) [34] (Fig. 10). In addition, ER stress also enhances DUSP6-induced gluconeogenesis by inducing PERK activation in the liver tissue of HFD-fed mice [161]. Collectively, DUSP6 dephosphorylates FOXO1 and induces FOXO1 nuclear translocation in the liver, leading to the enhancement of gluconeogenesis and glucose intolerance in mice.

**Fig. 11:**
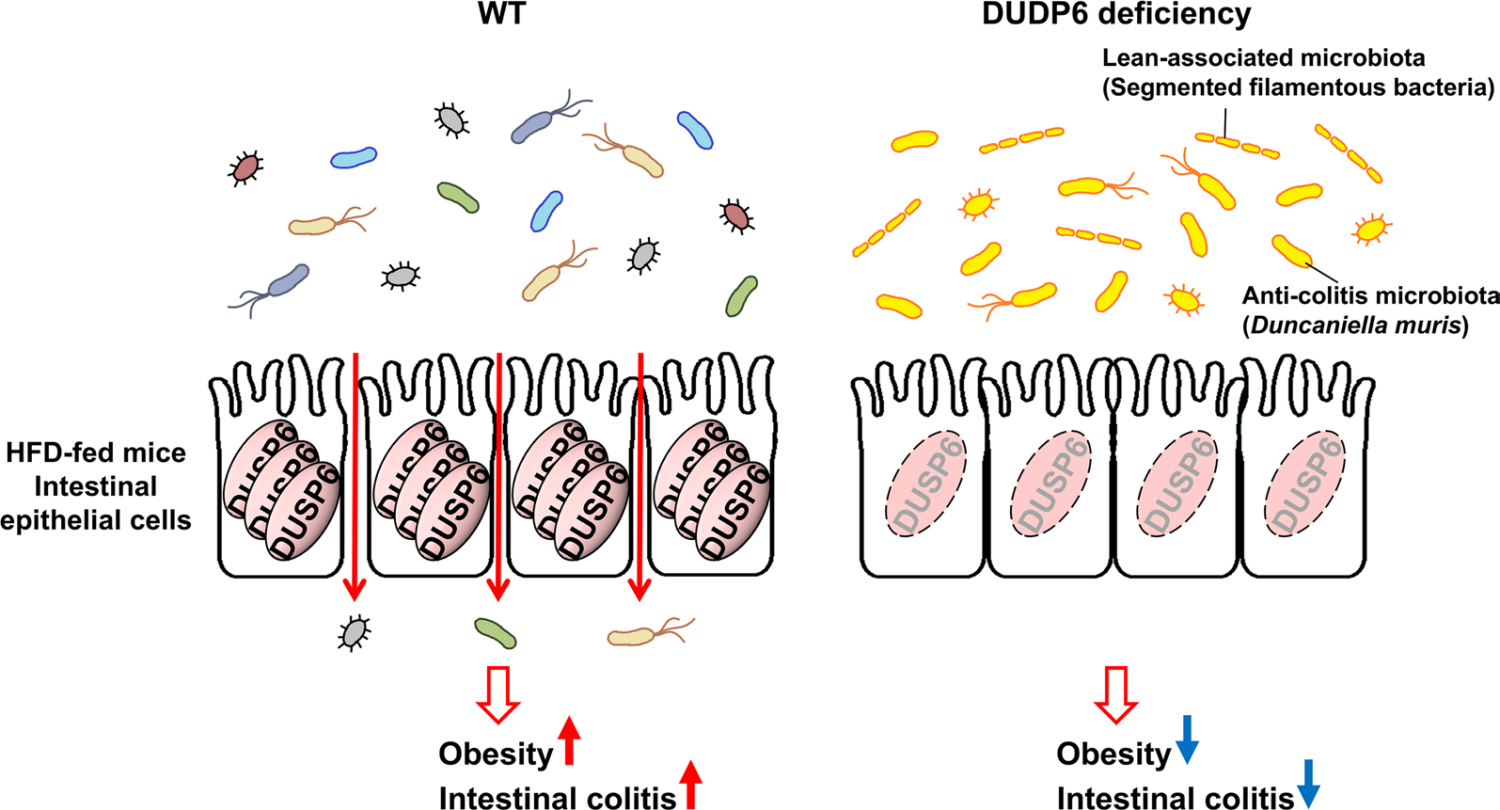
DUSP6 deficiency prevents HFD-induced obesity and intestinal colitis. HFD-fed WT mice show a downregulation of tight/adherens junction-related gene expression in intestinal epithelial cells, resulting in an increase of gut permeability and bacteria-induced intestinal colitis. In contrast, DUSP6-deficient mice show a resistance to HFD-induced obesity and intestinal colitis by increasing tight junction-related protein levels and altering gut microbiome in the intestine

DUSP6 deficient or KO mice are resistant to HFD-induced obesity [163–165]. Transplantation of fecal-microbiota from DUSP6 KO mice ameliorates diet-induced obesity in wild-type recipient mice, suggesting that DUSP6 deficiency results in an adjustment of intestine microbiome [164]. Similarly, DUSP6/DUSP8 double deficient mice are also resistant to HFD-induced obesity [166]. Due to the incomplete KO of DUSP8, the phenotype of double deficient mice may be a consequence of DUSP6 deficiency. Moreover, transcriptomics analysis of murine intestine shows that the adherens junction and tight junction pathways are downregulated by HFD feeding in wild-type mice, whereas these two pathways are upregulated by DUSP6 deficiency [164] (Fig. 11). The protein levels of the tight junction-related molecule ZO-1 are indeed increased in the intestine of HFD-fed DUSP6 KO mice compared to HFD-fed wild-type mice. Moreover, DUSP6 KO mice show a decrease of HFD-induced endotoxemia compared to wild-type mice. DUSP6 KO mice show a resistance to HFD-induced gut microbiome alteration [135, 164]; the decrease of symbiotic segmented filamentous bacteria population in the intestine of HFD-fed wild-type mice is suppressed by DUSP6 KO [164]. In summary, the increase of intestine barrier integrity by DUSP6 deficiency prevents HFD-induced gut microbiome alteration, as well as HFD-induced obesity.

HFD-fed DUSP6 homozygous KO mice display reduced hepatic lipid accumulation, hepatic steatosis, and NAFLD/NASH activity compared to those of DUSP6 wild-type and heterozygous KO mice [163]. Overexpression of cytochrome P450 4A (CYP4A), a fatty acid omega hydroxylase, contributes to the pathogenesis of metabolic liver steatosis [162, 167, 168]. CYP4A mRNA and protein levels are decreased in the hepatocytes of DUSP6 KO mice [163]. The palmitic acid and oleic acid-stimulated lipid accumulation is decreased by treatment of CYP4A inhibitor HET0016 in human HepG2 hepatoma cells [163]. These results suggest that DUSP6 may suppress CYP4A-mediated NAFLD.

DUSP6 protein levels are decreased in murine primary aortic endothelial cells derived from type 1 non-obese diabetic mice and high-glucose-treated human primary aortic endothelial cells [169]. Monocyte adhesion is an early event of diabetic cardiovascular inflammation and diabetic atherosclerosis [170, 171]. DUSP6 overexpression reduces high-glucose-induced monocyte adhesion in human primary aortic endothelial cells [169]. Treatment of sphingosine-1-phosphate (S1P) or S1P receptor agonists increases DUSP6 protein levels and reduces monocyte adhesion in the murine primary aortic endothelial cells. Conversely, treatment of S1P receptor antagonists decreases DUSP6 protein levels and induces monocyte adhesion [169]. These results suggest that dysregulation of the S1P-DUSP6 pathway may contribute to the pathogenesis of diabetic cardiovascular disease.

### DUSP7 (PYST2)

DUSP7 mRNA and protein levels are decreased in palmitate-treated murine primary hepatocytes, HFD-fed mice, ob/ob mice, simple steatosis patients, and NASH patients [172]. Moreover, DUSP7 protein levels are inversely correlated with NAFLD activity and serum levels of triglycerides, aspartate aminotransferase, and alanine aminotransferase in human participants. DUSP7 KO mice display increased HFD-induced body weight, liver mass, insulin resistance, hepatic lipid accumulation, hepatic steatosis, and NAFLD activity. Co-immunoprecipitation and GST pull-down assays show that DUSP7 interacts with TAK1. HFD-induced TAK1 activation is further enhanced by DUSP7 KO in the murine liver tissue. DUSP7 overexpression inhibits the lipid accumulation, proinflammatory cytokine mRNA levels, and TAK1 activation in palmitate-treated murine primary hepatocytes; the inhibited phenotypes are reversed by TAK1 overexpression [172]. Collectively, DUSP7 downregulation contributes to the pathogenesis of TAK1-mediated NAFLD.

### DUSP8 (M3/6)

Human DUSP8 is located near (downstream of) the T2D risk gene variant rs2334499 identified by GWAS [173, 174]. DUSP8 mRNA levels are elevated in the hypothalamus of HFD-induced diabetic mice and ob/ob mice, suggesting that DUSP8 may be involved in T2D [175]. DUSP8 KO mice display glucose intolerance and insulin resistance. Activation of the hypothalamic-pituitary-adrenal axis is enhanced in HFD-fed DUSP8 KO mice, resulting in hypercorticosteronemia. Conversely, DUSP8 overexpression by AAV viral transduction in the mediobasal hypothalamus of DUSP8 KO mice reverses the glucose intolerance to normal blood glucose levels. The role of DUSP8 in glucocorticoid signaling is further confirmed by neuron-specific DUSP8 cKO mice. DUSP8 cKO mice also display glucose intolerance and insulin resistance by HFD feeding [175]. These findings suggest that DUSP8 downregulates hypothalamic-pituitary-adrenal axis, resulting in the maintenance of glucose tolerance and insulin sensitivity. Mechanistically, overexpression of DUSP8 inhibits JNK activation and subsequently reduces glucocorticoid receptor phosphorylation of the inhibitory site Ser226 residue by JNK, resulting in the upregulation of glucocorticoid receptor activity and the downregulation of blood glucose levels [175]. The glucose intolerance and insulin resistance in DUSP8 whole-body KO mice are reversed by JNK1 whole-body KO in DUSP8/JNK1 dKO mice. Additionally, DUSP8 mRNA levels are increased in the hypothalamus of T2D patients compared to healthy individuals. Furthermore, the cerebral blood flow is higher in the hypothalamus of healthy volunteers carrying the T2D risk gene variant rs2334499 than volunteers carrying wild-type allele, indicating that the rs2334499 variant is positively correlated with insulin resistance [175]. Taken together, DUSP8 prevents insulin resistance by suppressing hypothalamic-pituitary-adrenal axis activation and promoting glucocorticoid receptor activity.

High-glucose (30 mM)-treated primary neonatal rat cardiac fibroblasts show an enhancement of cell proliferation and miR-21 expression, downregulating DUSP8 expression [176]. In contrast, DUSP8 protein levels are decreased in high-glucose-incubated primary neonatal rat cardiac fibroblasts. Reporter assays show that DUSP8 3′-UTR is targeted by miR-21 in primary rat cardiac fibroblasts. Overexpression of miR-21-mimic decreases DUSP8 protein levels, but increases cell proliferation and collagen synthesis, in high glucose-treated primary rat cardiac fibroblasts [176]. It remains to be studied whether high glucose induces miR-21 expression, leading to DUSP8 downregulation-induced diabetic cardiomyopathy.

### DUSP9 (MKP4)

DUSP9 (also named as MAPK phosphatase 4, MKP4) is a typical DUSP that inhibits the activation of ERK, p38, and JNK [151, 177–179]. DSUP9 is located near (upstream of) the T2D risk gene variant rs5945326 identified by GWAS [180, 181]. DUSP9 mRNA and protein levels are increased in insulin-stimulated murine adipocyte lines, as well as in the adipose tissue of HFD-induced obese mice, ob/ob mice, and db/db (*Lepr*^*db*^) mice [179, 182]. Adipocyte differentiation of murine 3T3-L1 cells is inhibited by DUSP9 overexpression [182]. Notably, overexpression of DUSP9 decreases blood glucose levels and improves glucose tolerance in ob/ob mice [179]. It is likely that DUSP9 is a negative regulator of adipocyte differentiation.

Gestational diabetes mellitus (GDM) occurs in pregnant women during the pregnancy and recovery after birth [183]. DUSP9 mRNA and protein levels are increased in the umbilical cord blood and placenta tissue of GDM patients compared to those of healthy pregnant women [184]. The induction of DUSP9 in GDM patients is positively correlated with fasting blood glucose and impaired glucose tolerance. Consistently, DUSP9 protein levels and lipid metabolism are increased in the murine GDM model. In addition, gluconeogenesis, insulin resistance, and placental cell apoptosis are induced, whereas β-cell function and insulin sensitivity are reduced in the GDM mice. Conversely, DUSP9 shRNA knockdown inhibits lipid metabolism, decreases blood glucose levels, enhances insulin sensitivity, and inhibits cell apoptosis in the GDM mice. DUSP9 directly dephosphorylates insulin receptor substrate 1 (IRS-1) at Tyr632 residue in human HTR-8/SVneo cells under high glucose stimulation. DUSP9 knockdown results in the induction of IRS-1 Tyr632 phosphorylation (stimulatory site) and subsequent activation of the downstream molecules phosphoinositide-3-kinase (PI3K)−AKT, promoting insulin sensitivity [184]. These results indicate that DUSP9 promotes the pathogenesis of GDM by downregulating IRS-1-PI3K-AKT signaling.

DUSP9 protein levels are decreased in the palmitate-treated murine primary hepatocytes and the liver tissue of HFD-fed mice and ob/ob mice [185]. Hepatocyte-specific DUSP9 cKO mice display the induction of body weight, liver mass, hepatic lipid accumulation, and NAFLD activity during HFD or high-fat-high-cholesterol (HFHC) diet feeding. Moreover, mRNA levels of NASH-related profibrotic genes are increased in the liver tissue of HFHC diet-fed of DUSP9 cKO mice. Mechanistically, DUSP9 inhibits apoptosis signal-regulating kinase 1 (ASK1) phosphorylation and subsequent ASK1-p38/JNK signaling in murine primary hepatocytes [185]. In summary, DUSP9 may prevent NAFLD/NASH by inhibiting ASK1-p38/JNK signaling.

### DUSP10 (MKP5)

DUSP10 (also named as MAPK phosphatase 5, MKP5) dephosphorylates JNK and p38 in vitro [186] and in African green monkey COS-7 kidney fibroblasts [187]. DUSP10 protein levels are decreased in the liver tissue of HFD-induced NAFLD mice; the DUSP10 downregulation is mediated by the E3 ubiquitin ligase TRIM7-induced K63-linked ubiquitination at Lys452 residue of DUSP10 and proteasomal degradation [188]. DUSP10 KO mice manifest an increase of body weight, fat mass, and insulin resistance [189, 190]. The insulin-stimulated AKT activation is reduced and the proinflammatory cytokines TNF-α/IL-6 are induced in the visceral adipose tissue of aged DUSP10 KO mice [189]. Furthermore, HFD-fed DUSP10 KO mice display enhanced hepatic steatosis and hepatic lipid accumulation compared to HFD-fed wild-type mice [190]. Mechanistically, DUSP10 inhibits p38 activation and consequently suppresses AFT2- and PPAR-γ-mediated transcription of cell death-inducing DFFA-like effector A (CIDEA) and cell death-inducing DFFA-like effector C (CIDEC) [190], which contribute to hepatic steatosis [191]. Taken together, DUSP10 downregulation contributes to fat accumulation, insulin resistance, and hepatic steatosis.

### DUSP12 (YVH1)

DUSP12 gene is located on human chromosome 1q23 locus, which is included in the T2D susceptibility gene locus 1q21-24 [192–194]. In this region, two T2D susceptibility single nucleotide polymorphisms (SNPs), rs1503814 [193] and rs1027702 [194], are identified to be at the upstream of the DUSP12 gene. DUSP12 protein levels, but not mRNA levels, are increased in the heart tissue of HFD-fed mice and in the primary neonatal rat ventricular myocytes (NRVMs) derived from high-glucose-treated rats [195]. DUSP12 deficiency results in the suppression of IRS1-AKT-GLUT4-mediated insulin signaling. DUSP12 deficiency also leads to the induction of oxidative stress and cell apoptosis in the heart tissue of HFD-fed mice and NRVMs of high-glucose-treated rats. Similarly, treatment of the ASK1 inhibitor GS-4997 reverses the induction of oxidative stress and cell apoptosis in DUSP12-deficient NRVMs of high-glucose-treated rats. Conversely, DUSP12 overexpression decreases oxidative stress and cell apoptosis in NRVMs of high-glucose-treated rats. In addition, DUSP12 deficiency induces myocardial hypertrophy, ventricular cavity enlargement, cardiac fibrosis, and cardiac dysfunction in HFD-fed mice, indicating that DUSP12 deficiency contributes to pathogenesis of diabetic cardiomyopathy. Mechanistically, DUSP12 binds to and dephosphorylates ASK1, resulting in the inactivation of ASK1 and its downstream molecules JNK and p38 [195]. These findings suggest that DUSP12 protects cardiomyocytes from oxidative stress and apoptosis by inhibiting ASK1-JNK/p38 signaling, leading to the suppression of diabetic cardiomyopathy pathogenesis.

DUSP12 protein levels, but not mRNA levels, are decreased in palmitic acid and oleic acid-treated human L02 hepatocytes, as well as in the liver tissue of HFD-induced obese mice and ob/ob mice [196]. Palmitic acid and oleic acid-induced lipid accumulation and proinflammatory cytokine production are increased by DUSP12 siRNA knockdown in human L02 hepatocytes. Similarly, HFD-fed hepatocyte-specific DUSP12 cKO mice display an induction of liver weight, gluconeogenesis, insulin resistance, hepatic lipid accumulation, and NAFLD activity. Notably, the body weight of DUSP12 cKO mice is increased by feeding with HFHC diet, but not HFD. Conversely, HFD-fed hepatocyte-specific DUSP12 transgenic mice show a reduction of liver weight, insulin resistance, hepatic lipid accumulation, and NAFLD activity. Moreover, overexpression of DUSP12 inhibits the activation of p38 and ASK1 in palmitic acid-treated human L02 hepatocytes. The induction of lipid accumulation and proinflammatory cytokine production by DUSP12 shRNA knockdown are reduced by overexpression of a dominant-negative ASK1 mutant in palmitic acid and oleic acid-treated human L02 hepatocytes [196]. These findings suggest that DUSP12 inhibits ASK1-mediated hepatic lipid accumulation and NAFLD.

### DUSP14 (MKP6)

DUSP14 mRNA and protein levels are increased in islet β-cells of glucagon-like peptide-1 (GLP-1)−treated mice [197]. GLP-1 is an incretin hormone that promotes murine β-cell proliferation through EGFR signaling [198]. Interestingly, knockdown of DUSP14 or overexpression of a dominant-negative DUSP14 mutant induces proliferation of murine islet β-cells [198]. These results suggest that DUSP14 may be involved in the negative regulation of β-cell proliferation.

DUSP14 protein levels are decreased in the palmitate or oleic acid-treated murine primary hepatocytes, as well as in the liver tissue of HFD-induced obese mice, ob/ob mice, and NAFLD patients [199]. HFD-fed hepatocyte-specific DUSP14 transgenic mice display a reduction of gluconeogenesis and insulin resistance, as well as the induction of glycogenesis in the liver tissue. Moreover, IRS-1−AKT signaling is enhanced in the liver tissue of HFD-fed hepatocyte-specific DUSP14 transgenic mice. Conversely, HFD-fed hepatocyte-specific DUSP14 cKO mice show an induction of gluconeogenesis and insulin resistance in the liver tissue. HFD-fed DUSP14 cKO mice also show an increase of liver weight, hepatic lipid accumulation, and proinflammatory cytokine production, whereas HDF-fed hepatocyte-specific DUSP14 transgenic mice do not. DUSP14 directly dephosphorylates TAB1 in TCR signaling, leading to inhibition of TAK1 activation and subsequent T-cell activation [29]. Consistently, TAK1 phosphorylation is decreased in the liver tissue of HFD-fed hepatocyte-specific DUSP14 transgenic mice and is increased in HFD-fed DUSP14 cKO mice [199]. Collectively, DUSP14 downregulation contributes to insulin resistance, hepatic lipid accumulation, and hepatic inflammation.

### DUSP15 (VHY)

DUSP15 (also named VH1-related member Y, VHY) is an atypical DUSP that suppresses the expression of myelin genes in the rat RT4 Schwann cell line [200]. Besides RT4 Schwann cells, DUSP15 mRNA and protein levels are decreased in the heart tissue of diabetic cardiomyopathy mice [201]. DUSP15 transgenic mice show a reduction of heart injury and an induction of heart function in HFD/streptozotocin-induced diabetic cardiomyopathy [202]. Mechanistically, DUSP15 overexpression increases mitochondrial HSP70 protein levels and then restores the downregulation of mitochondrial unfolded protein response in the heart tissue of diabetic cardiomyopathy mice [202]. It is still unclear whether DUSP15 directly dephosphorylates Thr116 residue on mitochondrial HSP70. Interestingly, molecular dynamics simulation analyses suggest that DUSP15 functions as a non-catalytic adaptor [203]. These results suggest that DUSP15 downregulation is associated with the pathogenesis of diabetic cardiomyopathy.

### DUSP16 (MKP7)

DUSP16 mRNA levels are decreased in the liver tissue of HFD-induced obese mice and ob/ob mice [204]. The lipid accumulation, proinflammatory cytokine production, and TAK1 activation are increased by DUSP16 siRNA knockdown in murine primary hepatocytes. Similarly, HFD-fed DUSP16-deficient mice display an enhancement of lipid synthesis, TAK1 activation, NF-κB signaling, and proinflammatory cytokine production in the liver tissue, as well as an induction of body weight and insulin resistance. Conversely, DUSP16 overexpression reduces lipid synthesis, JNK activation, TAK1 phosphorylation, NF-κB signaling, and proinflammatory cytokine production in palmitate-treated murine primary hepatocytes [204]. These findings implicate that DUSP16 may prevent the pathogenesis of obesity-induced hepatic steatosis.

### DUSP22 (JKAP, JSP1)

Diabetic nephropathy, as a common complication of T2D, is classified by irreversible fibrosis, excessive proliferation, and inflammation flare in kidney mesangial cells and glomerular basement membrane cells [205, 206]. DUSP22 mRNA and protein levels are decreased in high glucose (25 mM)-treated murine SV40-MES13 mesangial cells [205]. DUSP22 overexpression reduces cell proliferation, cell fibrosis, and proinflammatory cytokine production in high glucose-treated SV40-MES13 cells; conversely, these characteristics are increased by DUSP22 siRNA knockdown in low glucose (5.5 mM)-treated SV40-MES13 cells [205]. These results implicate that DUSP22 downregulation may promote diabetic nephropathy; however, the underlying mechanism remains unknown.

DUSP22 mRNA and protein levels are decreased in the liver tissue of NASH patients [207]. Moreover, hepatic DUSP22 protein levels are inversely correlated with NASH severity and NAFLD-associated hepatocellular carcinoma progression in patients. Similarly, hepatic DUSP22 protein levels are inversely correlated with NASH severity and NAFLD-associated HCC progression in patients. HFHC-fed hepatocyte-specific DUSP22 cKO mice display an induction of liver weight, insulin resistance, hepatic lipid accumulation, hepatic fibrosis, liver inflammation, and NAFLD activity. Moreover, HFHC-induced NF-κB signaling and MEK-ERK signaling in the murine liver tissue are further enhanced by DUSP22 cKO. Conversely, HFHC-fed hepatocyte-specific DUSP22 transgenic mice display opposite phenotypes compared to HFHC-fed DUSP22 cKO mice. In addition, DUSP22 dephosphorylates focal adhesion kinase (FAK) at Tyr397, Try576, and Tyr577 residues, leading to the inhibition of FAK inactivation and subsequent cell migration [208]. As expected, NASH patients show an increased FAK phosphorylation levels at Tyr397 and Tyr576/577 residues in their liver tissue [207]. Co-immunoprecipitation analyses and GST pull-down assays show that DUSP22 interacts with FAK. FAK activation is suppressed in the liver tissue of HFHC-fed hepatocyte-specific DUSP22 transgenic mice but is enhanced by hepatocyte-specific DUSP22 cKO. Consistently, HFHC-fed hepatocyte-specific FAK cKO mice display a reduction of liver weight, insulin resistance, hepatic lipid accumulation, liver damage, and proinflammatory cytokine production in HFHC-fed mice. Notably, injection of murine primary hepatocytes (overexpressing DUSP22 by AAV-transduction) can suppress HFHC diet-induced NAFLD phenotypes in wild-type mice. Taken together, DUSP22 downregulation promotes FAK-mediated NAFLD. DUSP22 *ex vivo* gene therapy may be a potential therapeutic strategy against NAFLD [207].

Interestingly, DUSP22 mRNA levels are significantly increased in the muscle of sarcopenia patients, leading to the disruption of myogenesis [209]. Treatment of the DUSP22 inhibitor BML-260 ameliorates skeletal muscle wasting in mice. It remains unclear whether a long-term treatment of BML-260 would induce NAFLD or autoimmune diseases.

### DUSP26 (MKP8, NEAP)

DUSP26 (also named mitogen-activated protein kinase phosphatase-8, MKP8, or neuroendocrine-associated phosphatase, NEAP [210]) mRNA levels are significantly downregulated in the pancreatic islet of diabetic db/db mice treated with the adipokine adipsin [211]. The adipsin treatment induces the production of C3a, an insulin secretagogue, and subsequent secretion of insulin [212]. Interestingly, cell apoptosis and DUSP26 mRNA levels are decreased by the co-treatment of C3a and palmitate in rat INS-1 β cells [211]. The cell apoptosis is increased by DUSP26 overexpression but decreased by DUSP26 shRNA knockdown or DUSP26 pharmacological inhibition in INS-1 β cells. The glycemia of db/db mice is decreased by the treatment of the DUSP26 inhibitor NSC-87877, which could improve the β-cell function of db/db mice [211]. Of note, the effect of the DUSP26 inhibitor on the insulin resistance of insulin-responsive tissues remains unclear. These results suggest that DUSP26 downregulation/inhibition may prevent T2D by preventing pancreatic β-cell apoptosis.

Different from the results of pancreatic cells, DUSP26 mRNA and protein levels are decreased in the heart tissue of diabetic db/db mice [213]. DUSP26 overexpression increases heart function and decreases myocardial fibrosis/hypertrophy in diabetic db/db mice. Moreover, DUSP26 overexpression increases ATP production and mitochondria fusion in the heart tissue of diabetic db/db mice, as well as decreases FAK and ERK activation. Kyoto Encyclopedia of Genes and Genomes (KEGG) pathway enrichment analysis shows that DUSP26 (possibly DUSP26 downregulation) is associated with FAK signaling in the palmitate-treated rat H9c2 cardiomyocytes under a high glucose (30 mM) condition. Treatment of the FAK agonist ZINC40099027 reverses DUSP26-inhibited ROS change, as well as reduces ATP production/mitochondria fusion in the palmitate-treated H9c2 cardiomyocytes under a high glucose condition [213]. These findings suggest that downregulation of DUSP26 may be involved in the pathogenesis of diabetic cardiomyopathy by inducing FAK signaling.

DUSP26 mRNA and protein levels are decreased in the kidney tissue of diabetic nephropathy patients [214] and acute kidney injury patients [215]. Methylation frequencies of the DUSP26 promoter regions are increased in the kidney tissue of acute kidney injury mice [215]. The kidney glomerular cell fibrosis, ROS production, MAPK activation, and nephropathy development of streptozotocin-induced diabetic mice are further enhanced by DUSP26 KO [214]. Mechanistically, DUSP26 suppresses cell apoptosis by dephosphorylating p53 at Ser312 residue [215]. Suppression of DUSP26 by shRNA knockdown or the DUSP26 inhibitor NSC87877 further enhances cell apoptosis in the kidney tissue of acute kidney injury mice. These results suggest that DUSP26 downregulation contributes to the pathogenesis of diabetic nephropathy and acute kidney injury.

DUSP26 protein levels are decreased in the liver tissue of ob/ob mice and HFD-induced obese mice [216]. HFD-fed hepatocyte-specific DUSP26 cKO mice display an induction of lipid accumulation, insulin resistance, gluconeogenesis, and liver damage; conversely, HFD-fed hepatocyte-specific DUSP26 transgenic mice show opposite phenotypes [216]. These results suggest that DUSP26 downregulation is involved in the pathogenesis of obesity-induced hepatic steatosis.

Taken together, DUSP26 contributes to T2D pathogenesis by inducing apoptosis of pancreatic β cells. In contrast, DUSP26 prevents diabetic cardiomyopathy, diabetic nephropathy, and obesity-induced hepatic steatosis. These contrasting results show that DUSP26 plays different roles in distinct organs; the underlying mechanisms remain unclear.

## The roles of DUSPs in cell signaling of cardiovascular diseases

Cardiovascular diseases are group of disorders affecting the heart and blood vessels [217], such as ischemic heart diseases and cardiomyopathy. Ischemic heart disease is primarily caused by atherosclerosis, which is a chronic inflammatory disease manifests plaque accumulation, vessel narrowing, and blood flow reduction [217, 218]. Cardiomyopathy is characterized by the altered size, shape, structure, and function of cardiac muscles, resulting in heart failure [217, 219]. Induction of several DUSPs, including DUSP3, DUSP6, DUSP7, DUSP8, and DUSP16, are involved in the pathogenesis of ischemic heart disease and cardiomyopathy (Table 7). In contrast, DUSP1, DUSP4, DUSP14, DUSP22, and DUSP26 are involved in the suppression of cardiomyopathy, ischemia/reperfusion (I/R) injury, and cardiac hypertrophy. In addition, DUSP6 deficiency contributes to the progression of diabetic cardiovascular disease, but to the suppression of myocardial infarction (MI). Similarly, downregulation of DUSP8 contributes to diabetic cardiomyopathy, but suppresses the pathogenesis of cardiac remodeling and cardiac hypertrophy. To date, the complex mechanisms of DUSP dysfunction in cardiovascular diseases are largely unclear.

### DUSP1 (MKP1, PTPN10) and DUSP4 (MKP2)

DUSP1 and DUSP4 protein levels are increased in the heart tissue of heart failure patients [220]. Similarly, DUSP1 and DUSP4 mRNA levels are increased in the heart tissue of cardiac hypertrophic mice, resulting in a decrease of p38 phosphorylation in the heart tissue [221, 222]. DUSP1/DUSP4 dKO mice, however, show a spontaneous development of cardiomyopathy, whereas individual DUSP1 and DUSP4 KO mice do not [222]. The activation of p38 is induced in the cardiomyocytes of symptomatic DUSP1/DUSP4 dKO mice. Notably, treatment with the p38 inhibitor SB731445 alleviates cardiomyopathy and restores cardiomyocyte contractility of DUSP1/DUSP4 dKO mice [222]. These results suggest that DUSP1 and DUSP4 may prevent cardiomyopathy by suppressing p38 activation.

### DUSP3 (VHR)

DUSP3 mRNA and protein levels are increased in the heart tissue of acute myocardial infarction (AMI) mice and hypoxia-stimulated murine primary neonatal cardiomyocytes [223]. The MI, cardiomyocyte apoptosis, and inflammatory cytokine production in AMI mice are reduced by DUSP3 shRNA knockdown. The levels of endothelial intercellular adhesion molecule 1 (ICAM-1)/vascular cell adhesion molecule 1 (VCAM-1), and activation of NF-κB are also reduced by DUSP3 shRNA knockdown in the heart tissue of AMI mice [223]. These results implicate that the DUSP3−NF-κB−ICAM/VCAM axis may be associated with AMI-induced heart injury.

### DUSP6 (MKP3)

DUSP6 KO mice display a decrease of TNF-α−stimulated ICAM-1 protein levels in the alveolus, aorta, and inferior vena cava [224]. DUSP6 KO mice also show a reduction of neutrophil infiltration in the lungs upon TNF-α or LPS challenge. Moreover, ICAM-1 protein levels are increased by DUSP6 overexpression but decreased by DUSP6 siRNA knockdown in human umbilical vein endothelial cells (HUVECs). The NF-κB−induced ICAM-1 promoter activity is also decreased by DUSP6 siRNA knockdown. In addition, the TNF-α−induced interaction between HUVECs and leukocytes is enhanced by DUSP6 overexpression [224]. These results suggest that DUSP6 induces NF-κB−promoted ICAM-1 transcription, contributing to vascular inflammation.

DUSP6 also contributes to heart injury in cardiac diseases. Myocardial infarcted DUSP6 KO rats and the DUSP6 inhibitor BPI-treated MI rats manifest an increase of cardiac function and survival rate, as well as a reduction of ischemia-induced cardiac fibrosis, cardiac infarction, and cardiomyocyte apoptosis [51, 225]. Like DUSP6 KO rats, cardiac hypertrophic mice show an improvement of heart function and a decrease of cardiac myocyte apoptosis by DUSP6 KO [226]. Notably, DUSP6 protein is stabilized by the deubiquitination enzyme OTUD1 in primary cardiomyocytes of MI mice [227]. In addition, DUSP6 mRNA and protein levels are increased in neutrophils and macrophages isolated from the peripheral blood and the heart tissue of MI rats [51]. Neutrophil-specific DUSP6 cKO mice display a decrease of ischemia-induced cardiac fibrosis, cardiac infarction, and cardiomyocyte apoptosis, as well as an improvement of cardiac function and post-MI survival rate. DUSP6-deficient neutrophils display enhanced ERK phosphorylation, suggesting that DUSP6 inhibits ERK activation. Interestingly, the transcription factor C/EBPβ binds to the DUSP6 promoter and promotes DUSP6 transcription. The binding of C/EBPβ to the DUSP6 promoter is blocked by treatment of the p38 inhibitor BIRB796 [51]. The enhancement of ERK activation by p38 inhibitor treatment may be due to the downregulation of C/EBPβ-induced DUSP6 transcription in neutrophils. Collectively, DUSP6 may be a potential therapeutic target for MI.

### DUSP7 (PYST2)

Cardiac-specific DUSP7 transgenic mice spontaneously develop dilated cardiomyopathy and display a shorter life span (10 months) [228]. The ERK activation but neither p38 nor JNK activation is drastically decreased in the heart tissue of cardiac-specific DUSP7 transgenic mice, whereas the levels of cardiomyopathy markers, atrial natriuretic peptide (ANP), and brain natriuretic peptide (BNP) are increased. Interestingly, treatment of the magnolia extract honokiol decreases ANP and BNP protein levels in the heart tissue of cardiac-specific DUSP7 transgenic mice [228]; however, ERK activation and DUSP7 protein levels are not changed. The mechanism of DUSP7-induced cardiomyopathy needs to be clarified.

### DUSP8 (M3/6)

DUSP8 mRNA levels are significantly increased in the heart tissue of dilated cardiomyopathy patients compared to those of healthy individuals [229, 230]. Similarly, DUSP8 protein levels are increased in the heart tissue of cardiac hypertrophic mice [230]. DUSP8-deficient mice display an increase of heart function and a decrease of hypertrophy marker β-myosin heavy chain (β-MHC) mRNA levels during the induction of cardiac hypertrophy. Conversely, heart-specific DUSP8 transgenic mice spontaneously display a reduction of heart function and an induction of cardiac hypertrophy. The activation of JNK, ERK, and p38 are inhibited in the heart tissue of heart-specific DUSP8 transgenic mice during the induction of cardiac hypertrophy [230]. These findings suggest that DUSP8 may contribute to the pathogenesis of cardiac hypertrophy.

### DUSP12 (YVH1)

DUS12 protein levels are decreased in the heart tissue of dilated cardiomyopathy patients [231]. Similarly, DUSP12 protein levels are decreased in angiotensin II (Ang II)-stimulated neonatal rat cardiomyocytes and the heart tissue of hypertrophy mice. Cardiac hypertrophic DUSP12 KO mice show enhanced disease symptoms, including a reduction of heart function and an induction of myocardial hypertrophy/fibrosis. Conversely, cardiomyocytes-specific DUSP12 overexpression attenuates myocardial hypertrophy/fibrosis and improves heart function in cardiac hypertrophic mice. Moreover, JNK activation is enhanced in the heart tissue of cardiac hypertrophic DUSP12 KO mice, but is suppressed in cardiac hypertrophic DUSP12 transgenic mice [231]. Treatment of the JNK inhibitor SP600125 ameliorates cardiac hypertrophy/fibrosis and improves heart function in cardiac hypertrophic DUSP12 KO mice. These findings suggest that DUSP12 downregulation-induced JNK activation may contribute to dilated cardiomyopathy.

DUSP12 protein levels are decreased in the myocardial tissue of rat I/R model [232]. DUSP12 overexpression suppresses cardiomyocyte apoptosis, oxidative stress, I/R injury, and myocardial tissue infarction of I/R rats. Mitophagy is a protective mechanism against I/R injury of cardiomyocytes [233]. DUSP12 overexpression increases protein levels of mitophagy-related molecules, such as HSPB8, in the myocardial tissue of I/R rats and H/R-stimulated rat H9c2 cardiac myoblasts [232]. Interestingly, co-immunoprecipitation analysis shows that DUSP12 binds to HSPB8 in H9c2 cardiac myoblasts. HSPB8 siRNA knockdown reverses the inhibition of cell apoptosis and oxidative stress in DUSP12-overexpressing H9c2 cells upon H/R stimulation [232]. These findings suggest that the DUSP12-HSPB8 axis may be involved in the pathogenesis of myocardial I/R injury.

DUSP12 mRNA levels are increased in the PBMCs or white-blood cells of patients with hypertension and left ventricular remodeling [234]. It remains unclear whether DUSP12 overexpression in immune cells contributes to the disease pathogenesis or induces a protective feedback mechanism. It would be interesting to study the roles of DSUP12 in individual immune cells in hypertension and left ventricular remodeling.

### DUSP14 (MKP6)

DUSP14 protein levels are decreased in the heart tissue of patients with dilated cardiomyopathy-induced heart failure [235]. Consistently, protein levels of DUSP14 and the cardiac hypertrophy markers ANP and β-MHC are decreased in the myocardium of cardiac hypertrophic mice and the Ang II-treated neonatal rat cardiomyocytes. After induction of cardiac hypertrophy by aortic banding, DUSP14 KO mice display an increase of cardiac hypertrophy and cardiac fibrosis, as well as a decrease of heart function. Conversely, cardiac-specific DUSP14 transgenic mice show an alleviation of cardiac hypertrophy. Furthermore, the activation of TAK1, JNK, and p38 in the heart tissue of hypertrophic mice is induced by DUSP14 KO, but reduced by cardiac-specific DUSP14 transgene. Treatment with the TAK1 inhibitor 5Z-7-ox suppresses cardiac fibrosis and enhances heart function in DUSP14 KO mice with cardiac hypertrophy [235]. These findings suggest that DUSP14 may prevent cardiac hypertrophy by inhibiting TAK1 activation.

Besides cardiac hypertrophy, DUSP14 protein levels are decreased in H/R-stimulated human AC16 cardiomyocytes [236] and in the heart tissue of mice with myocardial I/R injury [237]. Inflammatory cytokine production, MAPK activations, NF-κB signaling, and cell apoptosis are further enhanced by DUSP14 siRNA knockdown in H/R-stimulated murine primary cardiomyocytes or by DUSP14 KO in the heart tissue of I/R-injured mice [237]. H/R-stimulated ROS levels are increased in murine primary cardiomyocytes by DUSP14 siRNA knockdown. Treatment of the ROS inhibitor N-acetylcysteine suppresses MAPK activation, NF-κB signaling, cell apoptosis, and inflammatory cytokine production in H/R-stimulated murine primary cardiomyocytes [237]. These findings suggest that DUSP14 may prevent myocardial I/R injury by suppressing ROS-mediated NF-κB/MAPK activation.

In contrast to DUSP14 downregulation, miR-217 levels are increased in the heart tissue of myocardial I/R-induced mice [238], and miRNA-18a-5p levels are increased in H/R-stimulated human AC16 cardiomyocytes [239]. Murine DUSP14 mRNA 3’-UTR contains a miR-217-binding site [238] and a miR-18a-5p-binding site [236]. Overexpression of miR-217 mimic decreases DUSP14 mRNA/protein levels but increases MAPK/NF-κB activation in rat H9c2 cardiac myoblasts. Moreover, miR-217 mimic overexpression enhances cell apoptosis and inflammatory cytokine production in H/R-stimulated rat H9c2 cells, the enhanced phenotypes are abolished by DUSP14 overexpression or miR-217 antagonist treatment [238]. These findings suggest that miR-217-mediated DUSP14 downregulation may contribute to murine myocardial I/R injury. In addition, the cardiac miR-18a-5p binds to both DUSP14 3’-UTR and murine circular RNA APBB2 (circAPBB2, circ_0001352) [236]. The binding of miR-18a-5p to the DUSP14 3’-UTR is blocked by circAPBB2 overexpression, resulting in upregulation of DUSP14 protein levels [236, 239]. Furthermore, murine circAPBB2 overexpression or miRNA-18a-5p antagonist treatment induces DUSP14 production, resulting in the suppression of oxidative stress, cell apoptosis, and inflammatory cytokine production of H/R-stimulated AC16 cardiomyocytes. The suppression of cardiac injury is reversed by miRNA-18a-5p mimic overexpression or DUSP14 siRNA knockdown [236]. These results suggest that miRNA-18a-5p-mediated DUSP14 downregulation contributes to the progression of cardiomyocytes I/R injury. The results also implicate CircAPBB2 as a DUSP14 agonist by sponging miRNA-18a-5p, which downregulates DUSP14.

### DUSP16 (MKP7)

TNF-α increases DUSP16 protein levels and reduces JNK activation in HUVECs [240]. VCAM-1 contributes to monocyte adhesion, which is an early event of atherosclerosis [218, 241]. The nuclear IRF-1 protein levels, the binding of IRF-1 to the VCAM-1 promoter, and subsequent VCAM-1 transcription are all suppressed by DUSP16 siRNA knockdown in TNF-α-stimulated HUVECs [240]. The binding of IRF-1 to VCAM-1 is enhanced by overexpression of dominant-negative JNK1 mutant but reduced by overexpression of wild-type JNK1 in TNF-α-stimulated HUVECs [240]. These results suggest that DUSP16 may promote IRF-1−VCAM-1−mediated atherosclerosis.

### DUSP22 (JKAP, JSP1)

Serum DUSP22 levels are decreased in patients with coronary heart disease (CHD) [242]. Moreover, serum DUSP22 levels are inversely correlated with coronary artery stenosis, Th1/Th17 populations, and serum levels of CRP, IFN-γ, and TNF-α in CHD patients. Th1- and Th17-mediated chronic inflammation and plaque formation contribute to the development of atherosclerosis, leading to CHD [243, 244]. Th1/Th17 differentiation is enhanced by DUSP22 siRNA knockdown and is suppressed by DUSP22 overexpression in human primary naïve CD4^+^ T cells derived from CHD patients [242]. Notably, the Th1/Th17 differentiation of DUSP22 siRNA knockdown T cells is reduced by treatment of the MEK (ERK activator) inhibitor PD98059 or the NF-κB inhibitor BAY-11-7082. In addition, HFD-fed T-cell-specific DUSP22 cKO mice display an induction of atherosclerosis, ERK/NF-κB activation, and Th1/Th17 populations in the aorta compared to HFD-fed wild-type mice [242]. These findings show that DUSP22 downregulation enhances Th1/Th17 differentiation, contributing to the progression of CHD.

Unlike attenuation of inflammation by DUSP22 in CHD, DUSP22 contributes to neutrophil-driven vascular inflammation. DUSP22 knockout results in a reduction of vascular hemorrhage in the skin tissue of LPS plus TNF-α−stimulated mice [245]. In addition, LPS plus TNF-α−stimulated skin vascular hemorrhage in wild-type mice is ameliorated by the adoptive transfer of DUSP22 KO. These findings suggest that DUSP22 is involved in the pathogenesis of neutrophil-mediated vascular hemorrhage.

### DUSP26 (MKP8, NEAP)

DUSP26 mRNA and protein levels are increased in the heart tissue of cardiac hypertrophy mice [246]. Similarly, DUSP26 protein levels are increased in the Ang II-treated rat H9c2 cardiomyocytes. Interestingly, mRNA levels of cardiac hypertrophy markers, such as ANP and β-MHC, are further increased by DUSP26 siRNA knockdown in Ang II-treated rat H9c2 cardiomyocytes, but decreased by DUSP26 overexpression. Moreover, TAK1 phosphorylation at Thr187 residue is reduced by DUSP26 overexpression in Ang II-treated rat H9c2 cardiomyocytes. Heart-specific DUSP26 cKO mice display enhanced the activation of TAK1, JNK, and p38, as well as the induction of cardiac hypertrophy and fibrosis upon aortic constriction. Conversely, DUSP26 overexpression attenuates the symptoms in cardiac hypertrophic wild-type mice. Treatment of the TAK1 inhibitor 5Z-7-ox also reverses disease symptoms in cardiac hypertrophic heart of DUSP26 cKO mice [246]. These findings suggest that DUSP26 protects mice from cardiac hypertrophy by suppressing TAK1-JNK/p38 signaling.

## The roles of DUSPs in cell signaling of other chronic diseases

The DUSP family members are involved in the development of other chronic bone diseases such as osteoarthritis and osteoporosis. Understanding the signal pathways of DUSPs, such as DUSP3 and DUSP14 in these diseases would be helpful for developing therapeutic strategies against chronic diseases.

### DUSP3 (VHR)

DUSP3 mRNA and protein levels are increased in the kidney tissue of mice with renal I/R [247]. DUSP3 KO mice display enhanced renal function and renal angiogenesis, as well as reduced inflammatory cytokine production and renal injury after renal I/R induction compared to those of wild-type mice [247]. These results show that DUSP3 may be involved in the pathogenesis of renal I/R injury.

### DUSP14 (MKP6)

DUSP14 mRNA and protein levels are decreased in IL-1β−stimulated rat primary chondrocytes, in murine differentiated osteoclasts and the articular cartilage tissue of osteoarthritis rats [248, 249]. Conversely, DUSP14 overexpression reduces articular cartilage damage and disease severity in osteoarthritis rat [248]. Moreover, DUSP14 overexpression enhances AMPK activation and suppresses apoptosis, NF-κB signaling, and pro-inflammatory cytokine production in IL-1β-stimulated rat primary chondrocytes. The suppression of cell apoptosis and pro-inflammatory cytokine production by DUSP14 is reversed by AMPK siRNA knockdown [248]. DUSP14 overexpression suppresses pro-inflammatory cytokine mRNA expression, NF-κB signaling, and cell apoptosis during in vitro murine osteoclast differentiation [249]. Moreover, DUSP14 transgenic mice display an alleviation of bone loss during the induction of inflammatory osteoporosis, whereas DUSP14-deficient mice display an induction of inflammation responses and osteoclast apoptosis [249]. These findings show that DUSP14 downregulation is associated with the progression of osteoarthritis and inflammatory osteoporosis.

DUSP14 protein levels are decreased in the liver tissue of patients with NAFLD [199], which is associated with osteoporosis in patients [250, 251]. Osteoprotegerin (OPG), secreted by multiple tissues (such as bone, heart, and liver), suppressing osteoclast differentiation and subsequent osteoporosis [252–254], is downregulated in the liver tissue of NASH patients [252]. Interestingly, DUSP14 mRNA and protein levels are also decreased in the liver tissue of OPG KO mice fed with methionine and choline-deficient diet compared to those of wild-type mice fed with the same diet [252]. Conversely, OPG overexpression restores the DUSP14 protein levels in palmitic acid-treated human L02 hepatocytes. These results implicate that DUSP14 downregulation may be involved in the NASH-associated osteoporosis.

## DUSPs in tumorigenesis, metastasis, and therapy resistance

The roles of DUSPs in tumorigenesis and tumor metastasis have been extensively reviewed (reviewed in [255–271]). Upregulation of DUSP4 [255], DUSP8 [272–274], DUSP12 [275, 276], DUSP23 [277], and DUSP24 [278, 279], as well as downregulation of DUSP2 [255] and DUSP22 [207, 280–282] contribute to tumorigenesis and tumor progression. DUSP1, DUSP3, DUSP6, DUSP7, DUSP9, DUSP10, DUSP14, DUSP16, and DUSP26 are also associated with tumorigenesis [69, 153, 240, 255, 267–270, 283–298]. For tumor metastasis, the enhancement of DUSP6 [255], DUSP8 [272–274], DUSP11 [299], DUSP24 [279], and DUSP28 [300], as well as the suppression of DUSP1 [257], DUSP3 [301–303], DUSP14 [304], DUSP16 [305], and DUSP22 [208] play critical roles in the migration and metastasis of tumor cells. The roles of DUSPs in cancer drug resistance have also been widely studied (reviewed in [255]). DUSP1 [255, 271], DUSP5 [271], DUSP6 [255], DUSP7 [306–309], DUSP14 [271], DUSP16 [255], DUSP26 [310], and DUSP28 [300] are increased but DUSP2 [255] is decreased in patients that are resistant to cancer therapy. DUSP4, DUSP8, DUSP9, and DUSP10 are also associated with cancer therapy resistance [255, 271, 274, 291]. Thus, multiple DUSPs and their signaling molecules could be potential biomarkers or therapeutic targets of human cancers. Furthermore, several DUSPs (DUSP2, DUSP6, DUSP9, and DUSP22) are potential targets for cancer immunotherapies [282, 311–313]. The complex regulatory networks of DUSP dysregulation in various human cancers still need to be further elucidated.

## Discussion and conclusion

Individual DUSPs family members are involved in regulations of multiple physiological responses, such as tissue inflammation, cell apoptosis, cell proliferation, and cell differentiation. Many DUSP proteins display structural similarity and share overlapped MAPK substrates. Functional redundancy of multiple DUSPs has been a widely discussed topic [28, 222, 314, 315]; however, it is not supported by the phenotypes or symptoms of individual DUSP KO murine lines in the presence of other DUSPs. For example, DUSP1 KO mice display accelerated arthritis symptoms upon the collagen immunization [56, 59]. DUSP2 KO mice display exacerbated colitis symptoms after the DSS administration [33]. DUSP11-deficient mice display an enhancement of LPS-induced proinflammatory cytokine production and endotoxic shock [95]. DUSP14-deficient mice show enhanced T-cell activation and display severe symptoms during the induction of EAE [29]. DUSP22 KO mice spontaneously develop multi-organ inflammation [31, 77] and AS-like symptoms [77]. Among 26 DUSP phosphatases, dysregulation of 19 DUSPs is associated with the pathogenesis of inflammatory diseases, chronic diseases, or cancers. The roles of the remaining 6 DUSPs, DUSP13/DUSP18/DUSP19/DUSP21/DUSP29, need to be investigated in the future. Interestingly, these remaining 6 DUSPs are atypical DUSPs lacking the KIM; therefore, these DUSPs may bind to and regulate non-MAPK molecules. Notably, typical DUSPs (MKPs) also interact with and dephosphorylate non- MAPK substrates. For example, DUSP6 dephosphorylates the transcription factor FOXO1, promoting *PEPCK* and *G6Pase* transcription [34]. DUSP9 dephosphorylates IRS-1 and inhibits insulin signaling [184]. These practical evidences all point out that individual DUSPs play distinct and non-redundant functional roles in various signaling pathways and disease pathogenesis. Nonetheless, functional interactions amongst different DUSPs may contribute to a single disease.

In individual inflammatory diseases, every DUSP may show distinct functions in different tissues and cell types. For example, DUSP1 is decreased in the symptomatic tissues of psoriasis and glomerulonephritis patients [62–64], but is increased in the heart tissue of heart failure patients [220]. DUSP14 is induced in peripheral blood T cells of AS patients [77], but is reduced in the liver tissue of NAFLD patients [199] and the heart tissue of heart failure patients [235]. In addition, DUSP9 is induced in the umbilical cord blood and placenta tissue of GDM patients with insulin resistance [184], but is reduced in the liver tissue of HFD-fed mice and ob/ob mice with insulin resistance [185]. These findings suggest that a single DUSP molecule (either typical or atypical DUSP) may target different tissue-specific substrates, regulating distinct cell signaling pathways in different tissues. Furthermore, both typical (MKPs) and atypical DUSPs dephosphorylate/target either MAPKs or non-MAPK substrates, resulting in the diversity of pathophysiological responses by typical/atypical DUSP dysregulation. It would be valuable to investigate the distinct functions of DUSPs in various tissues of inflammatory and chronic diseases using tissue-specific KO animals.

Overexpression of DUSP1, DUSP3, DUSP4, DUSP5, DUSP6, DUSP7, DUSP8, DUSP9, DUSP14, DUSP23, and DUSP26 may contribute to the pathogenesis of AS, cardiac hypertrophy, AMI, IBD, obesity, diabetes, NAFLD, or cancer, suggesting that these DUSPs are potential therapeutic targets for inflammatory/chronic diseases. Notably, treatment with various DUSPs’ small molecule inhibitors reduces inflammatory response in cell lines and preclinical animal disease models [255, 316]. In contrast, DUSP overexpression alleviates pathological symptoms in animal models of DUSP downregulation-induced diseases. For example, DUSP2 overexpression mitigates nephritis in MRL/lpr mice [66]. DUSP5 overexpression attenuates the induced CIA in mice [80]. DUSP14 overexpression suppresses Th2-mediated allergic asthma [131], cardiac hypertrophy [235], and osteoarthritis in animal models [248]. Finally, overexpression of DUSP26 inhibits diabetic cardiomyopathy in mice [213]. To date, developments of specific agonists or activators of individual DUSPs are challenging. It would be important to study the regulatory mechanisms of DUSP in human diseases. For example, upstream regulators of transcriptional regulation and post-transcriptional modifications that downregulate DUSPs would be potential targets for the diseases. Taken together, understanding the regulatory mechanisms and DUSP signaling pathways will provide new directions for formulating therapeutic strategies to inflammatory/chronic diseases.

## Data Availability

No datasets were generated or analysed during the current study.

## Abbreviations

3′-UTR: 3′-Untranslated region
AAV: Adeno-associated virus
AKT: Protein kinase B
AMI: Acute myocardial infarction
Ang II: Angiotensin II
ANP: Atrial natriuretic peptide
AS: Ankylosing spondylitis
ASK1: Apoptosis signal-regulating kinase 1
BASDAI: Bath Ankylosing Spondylitis Disease Activity Index
BNP: Brain natriuretic peptide
BUN: Blood urea nitrogen
CD: Crohn’s disease
CDAA: Choline-deficient L-amino acid-defined
CHD: Coronary heart disease
CIA: Collagen-induced arthritis
CIDEA: Cell death-inducing DFFA-like effector A
CIDEC: Cell death-inducing DFFA-like effector C
cKO: Conditional knockout
CYP4A: Cytochrome P450 4A
dKO: Double knockout
DSP: Dual-specificity phosphatase (used for family name)
DSS: Dextran sulfate sodium
DUSP: Dual-specificity phosphatase (used for subgroup name)
EAE: Experimental autoimmune encephalomyelitis
EMT: Epithelial-to-mesenchymal transition
ERK: Extracellular regulated kinase
FAK: Focal adhesion kinase
G6Pase: Glucose 6-phosphatase
GDM: Gestational diabetes mellitus
GLP-1: Glucagon-like peptide-1
GWAS: Genome-wide association study
H/R: Hypoxia/reoxygenation
HDAC3: Histone deacetylase 3
HDL: High-density lipoprotein
HFD: High-fat diet
HFHC: High-fat-high-cholesterol
HUVEC: Human umbilical vein endothelial cell
I/R: Ischemia/reperfusion
IBD: Inflammatory bowel disease
ICAM-1: Intercellular adhesion molecule 1
IGF-1: Insulin-like growth factor 1
IRS-1: Insulin receptor substrate 1
JNK: C-Jun N-terminal kinase
KEGG: Kyoto Encyclopedia of Genes and Genomes
KIM: Kinase-interacting motif
KO: Knockout
LN: Lupus nephritis
LPS: Lipopolysaccharide
MAPK: Mitogen-activated protein kinase
MAS: Multiple autoimmune syndrome
MI: Myocardial infarction
MKP: MAP kinase phosphatase
MTM: Myotubularin
NAFLD: Non-alcoholic fatty liver disease
NASH: Non-alcoholic steatohepatitis
NRVM: Neonatal rat ventricular myocyte
OPG: Osteoprotegerin
PBMC: Peripheral blood mononuclear cell
PGC-1α: PPAR-γ coactivator 1 α
PI3K: Phosphoinositide-3-kinase
PTP: Protein tyrosine phosphatase
RA: Rheumatoid arthritis
S1P: Sphingosine-1-phosphate
scRNA-seq: Single-cell RNA sequencing
SLE: Systemic lupus erythematosus
SNP: Single nucleotide polymorphism
SS: Sjögren's syndrome
SSH: Slingshot
T2D: Type 2 diabetes
TAK1: TGF-β-activated kinase 1
UC: Ulcerative colitis
VCAM-1: Vascular cell adhesion molecule 1
β-MHC: β-Myosin heavy chain
